# Potential distribution of fall armyworm in Africa and beyond, considering climate change and irrigation patterns

**DOI:** 10.1038/s41598-021-04369-3

**Published:** 2022-01-11

**Authors:** Bipana Paudel Timilsena, Saliou Niassy, Emily Kimathi, Elfatih M. Abdel-Rahman, Irmgard Seidl-Adams, Mark Wamalwa, Henri E. Z. Tonnang, Sunday Ekesi, David P. Hughes, Edwin G. Rajotte, Sevgan Subramanian

**Affiliations:** 1 Chemical Ecology Lab, Department of Entomology, The Pennsylvania State University, Orchard Road, University Park, PA 16802 USA; 2 International Center for Insect Physiology and Chemical Ecology, Nairobi, Kenya

**Keywords:** Ecology, Climate-change ecology, Phenology

## Abstract

The fall armyworm, *Spodoptera frugiperda* (FAW), first invaded Africa in 2016 and has since become established in many areas across the continent where it poses a serious threat to food and nutrition security. We re-parameterized the existing CLIMEX model to assess the FAW global invasion threat, emphasizing the risk of transient and permanent population establishment in Africa under current and projected future climates, considering irrigation patterns. FAW can establish itself in almost all countries in eastern and central Africa and a large part of western Africa under the current climate. Climatic barriers, such as heat and dry stresses, may limit the spread of FAW to North and South Africa. Future projections suggest that FAW invasive range will retract from both northern and southern regions towards the equator. However, a large area in eastern and central Africa is projected to have an optimal climate for FAW persistence. These areas will serve as FAW ‘hotspots’ from where it may migrate to the north and south during favorable seasons and then pose an economic threat. Our projections can be used to identify countries at risk for permanent and transient FAW-population establishment and inform timely integrated pest management interventions under present and future climate in Africa.

## Introduction

The fall armyworm (FAW), *Spodoptera frugiperda* J.E. Smith (Lepidoptera: Noctuidae), is an insect pest native to the Americas that has invaded and spread throughout sub-Saharan Africa within the last four years^1^. Since its introduction, FAW has emerged as a serious threat to cereal crops' productivity, such as maize and sorghum, two of the major staple food crops of smallholder farmers, thus jeopardizing food security throughout Africa^2,3^. In order to contain the FAW spread, many African countries have distributed and applied synthetic pesticides. In 2017, Zimbabwe distributed nearly 102,000 L of pesticide valued at 1.97 million US dollars to farmers^4^. Although subsidized by governments, the use of synthetic pesticides as the sole control measure is unsustainable due to their high cost, risk of increased pesticide resistance, pest resurgence, and risk to human health and the environment^5^. Hence, there is a need to develop new, more sustainable approaches to FAW management. Alternative control measures are being considered including the use of biopesticides^6^, biological control^7–9^ , and agroecological practices such as intercropping with legumes, push–pull system, and diversifying the farm environment^10–12^. The choice and scaling of these alternative solutions depend on accurate characterization of the potential permanent and temporal distribution of FAW across the continent as well as how FAW and the agriculture production system will respond to a changing climate. Further, such information will refine our understanding of the pest’s potential migratory behavior in Africa, and is essential for developing long-term strategies to mitigate FAW infestations in hotspots (regions where FAW is endemic) as well as transient areas where FAW may move from hotspots to occupy a region for a period of favorable climate conditions.

FAW is a polyphagous pest with a wide host range, feeding on 353 plant species, including many food crops, forages and turfgrass^13,14^. The most affected crops in the Americas, the native range of FAW, are maize and sorghum^13^. FAW larvae have voracious appetites and cause severe damage to plants^15^. The female FAW moth lays eggs on the distal part of the maize leaf. Upon hatching the first and second instar larvae feed on that leaf, but eventually they enter the leaf whorl and feed on the unfurling leaves causing extensive defoliation. As plants mature, FAW larvae often start feeding on the ear. Finally, the larvae pupate in the soil^16^. Pupation lasts 8–30 days until the adults emerge^17^. Under ideal conditions, adult moths live up to 14 days, capable of migrating to distant new areas. In warm climate, FAW completes its entire life cycle in 3–4 weeks, but in cold climate, it takes considerably longer^17^. Unlike other lepidopteran pests, FAW cannot survive in areas with extended freezing temperatures. It lacks any diapause mechanisms and overwinters only in warm and moist areas^14^. Although its year-round distribution is restricted to tropical and subtropical regions, adult moths can migrate substantial distances into temperate regions during warm summers^14^ and establish transient populations which can jeopardize harvests in these regions. If left unchecked, FAW in Africa has the potential to cause an economic loss of around 13 billion US dollars per year through damage to maize, sorghum, rice, and sugarcane, alone^15^.

Outside of the native range in North and South America, FAW was first reported in Nigeria, São Tomé and Príncipe in early 2016^1^, from where it is believed to have spread to other African countries, including Kenya, Uganda, Rwanda, Ethiopia, and Tanzania. By April 2018, FAW had invaded and spread throughout sub-Saharan Africa and Sudan^18^. Recently, FAW has been reported in Egypt^19^. The presence of FAW in North Africa substantially increases the risk of FAW invasion of Europe through migration. Beyond Africa, FAW has also invaded several countries in Asia^20–23^ and Australia^24^. The rapid spread of FAW across the world may be due to trade and weak phytosanitary regulations, but also due to migratory behavior of the pest itself^25–28^.

One of the major determining factors affecting pest populations and their distribution is climate, which may either directly affect the physiology of the pest or indirectly impact parasitoids or predators, competitors, and food sources^29–31^. If global temperatures increase as projected, multivoltine migratory insects such as FAW could increase their number of generations per year^32^ and expand their infestation range to higher latitudes and elevations. As FAW is rapidly spreading across Africa and beyond, we urgently need information about its potential invasion and establishment threat in the present and possible future climates.

Species distribution modeling is an effective method of predicting pest invasion risk and establishment potential in the region of focus. The correlative model MaxEnt and the process-based semi-mechanistic model CLIMEX are the two most popular models used to assess the risk of FAW invasion and establishment^33–41^. Using the CLIMEX model, Ramirez-Cabral et al*.*^38^ assessed the possible FAW distribution changes in its native ranges under climate change scenarios. This model did not distinguish between permanent and transient FAW populations. It projected optimal climatic suitability in northeastern states of the USA. Later, Du Plessis et al*.*^35^ reparameterized the CLIMEX model and identified the FAW potential invasion and establishment areas under the historical climate conditions broadly. However, the pest's potential distribution in Africa, the most affected continent, under future climate conditions was not projected. Yet, the knowledge of areas of habitat suitability for transient, migrant and permanent FAW populations under climate change scenarios, including irrigation pattern is necessary to enable policymakers at the national and regional levels to develop sustainable management strategies and to identify research priorities under the present and future climates in Africa and beyond. This study aims to fill this knowledge gap.

We used CLIMEX 4.0^42^, combined with eco-physiological tolerances and global occurrence records, to assess and predict the FAW invasion and establishment risk under both historical and future climate conditions, taking also the effect of irrigation into account. In addition, we performed the sensitivity analysis to test the effect of parameter changes on the modeling outcomes and identify the parameters significantly influencing the CLIMEX results for the distribution of FAW. By mapping where transient and permanent FAW populations might potentially become established, the results from this paper can support strategic decision-making.

## Methods

### Research model and software

#### CLIMEX model

FAW growth and development are primarily related to climate conditions, especially temperature patterns^17^. The current study used CLIMEX (version 4)^42^, a semi-mechanistic niche modeling platform, to project FAW distribution in relation to climate. The model parameters that describe the species’ response to climate were overlaid onto FAW occurrence data and climate data to project the species’ potential global distribution. Briefly, the annual growth index (GI) was used to describe the potential for FAW population growth during favorable climatic conditions, while stress indices (SI: cold, wet, hot, and dry) and interaction stresses (SX: hot-dry, hot-wet, cold-dry, and cold-wet) (Table 1) were applied to describe the probability that FAW populations could survive unfavorable conditions. The Ecoclimatic index (EI) was derived from a combination of GI, SI, and SX indices to provide an overall annual index of climatic suitability on a scale of 0–100^42^. An EI value of 0 indicates that the location is not suitable for the long-term survival of the species, whereas an EI value of 100 indicates maximum climatic suitability comparable to conditions in incubators. EI values of more than 30 indicate the optimal climate for a species. In this study, the climatic suitability was classified into four arbitrary categories; unsuitable for EI = 0, marginal for 0 < EI ≤ 10, suitable for 10 < EI ≤ 30, and optimal for 30 < EI ≤ 100^42^.

**Table 1:** CLIMEX parameter values used for modeling the distribution and invasion risk of FAW (*Spodoptera frugiperda*).

Parameters		Ramirez-Cabral et al. 2017	Du Plessis et al. 2018	Current study
**Temperature**				
DV0	Lower temperature threshold (°C)	12	12	12
DV1	Lower optimal temperature (°C)	22	25	25
DV2	Upper optimal temperature (°C)	27	30	30
DV3	Upper temperature threshold (°C)	34	39	**36**
**Soil moisture**				
SM0	Lower soil moisture threshold	0.1	0.15	0.15
SM1	Lower optimal soil moisture	0.7	0.8	0.8
SM2	Upper optimal soil moisture	0.9	1.5	1.5
SM3	Upper soil moisture threshold	1.5	2.5	**2.0**
**Cold stress**				
TTCS	Cold stress temperature threshold (°C)	8	12	**8**
THCS	Cold stress accumulation rate (week^−1^)	− 0.00001	0.001	− **0.005**
**Heat stress**				
TTHS	Heat stress temperature threshold (°C)	38	39	39
THHS	Heat stress accumulation rate (week^−1^)	0.001	0.005	**0.0025**
**Dry stress**				
SMDS	Soil moisture dry stress threshold	0.1	0.1	0.1
HDS	Dry stress accumulation rate (week^−1^)	− 0.001	− 0.005	− 0.005
**Wet stress**				
SMWS	Soil moisture wet stress threshold	1.5	2.5	2
HWS	Wet stress accumulation rate (week^−1^)	0.001	0.002	**0.01**
**Threshold annual heat sum**				
PDD	Minimum degree day sum needed to complete a generation	559	600	**400**
**Irrigation** (mm day^−1^)		No	2.5	2.5

Changes made to the Du Plessis et al.^35^ parameter values are given in bold.

#### ArcGIS software

The ArcGIS software 10.8 (US Environment Systems Research Institute—ESRI, Redlands, CA, USA, https://desktop.arcgis.com/en/arcmap/) was used to visualize the result obtained from the CLIMEX analysis and calculate the areas under various EI categories for the species.

### Data collection

#### Fall armyworm occurrence data

FAW occurrence observations (n = 304) within its native range were obtained from the Global Biodiversity Information Facility (GBIF, www.gbif.org), PestWatch (www.pestwatch.psu.edu), Butterflies and Moths of North America (BAMONA, www.butterfliesandmoths.org), and literature resources (Supplementary Table S1). Real-time occurrence records (n = 1186) in six East African countries (Burundi, Ethiopia, Kenya, Rwanda, Tanzania, and Uganda) were collected from the Community Based FAW monitoring forecasting and Early Warning (CBFAMFEW) system^44,45^. The CBFAMFEW system relies on pheromone traps, field scouting and mobile applications for field data collection. Briefly, FAW pheromone traps were established in five districts in each country, and, in each district, 10 villages were sampled under the coordination of two community focal persons. Thus collected FAW occurrence records were validated by national FAW focal persons and published in FAMEWS (Fall Armyworm Monitoring and Early Warning System) global platform^44,45^.

The additional records of FAW occurrence were obtained from PlantVillage FAMEWS survey. PlantVillage is a public good platform that integrates AI, satellites, cloud computing, and local networks to help smallholder farmers adapt to climate changes and increased pest pressure.

**Figure 1:**
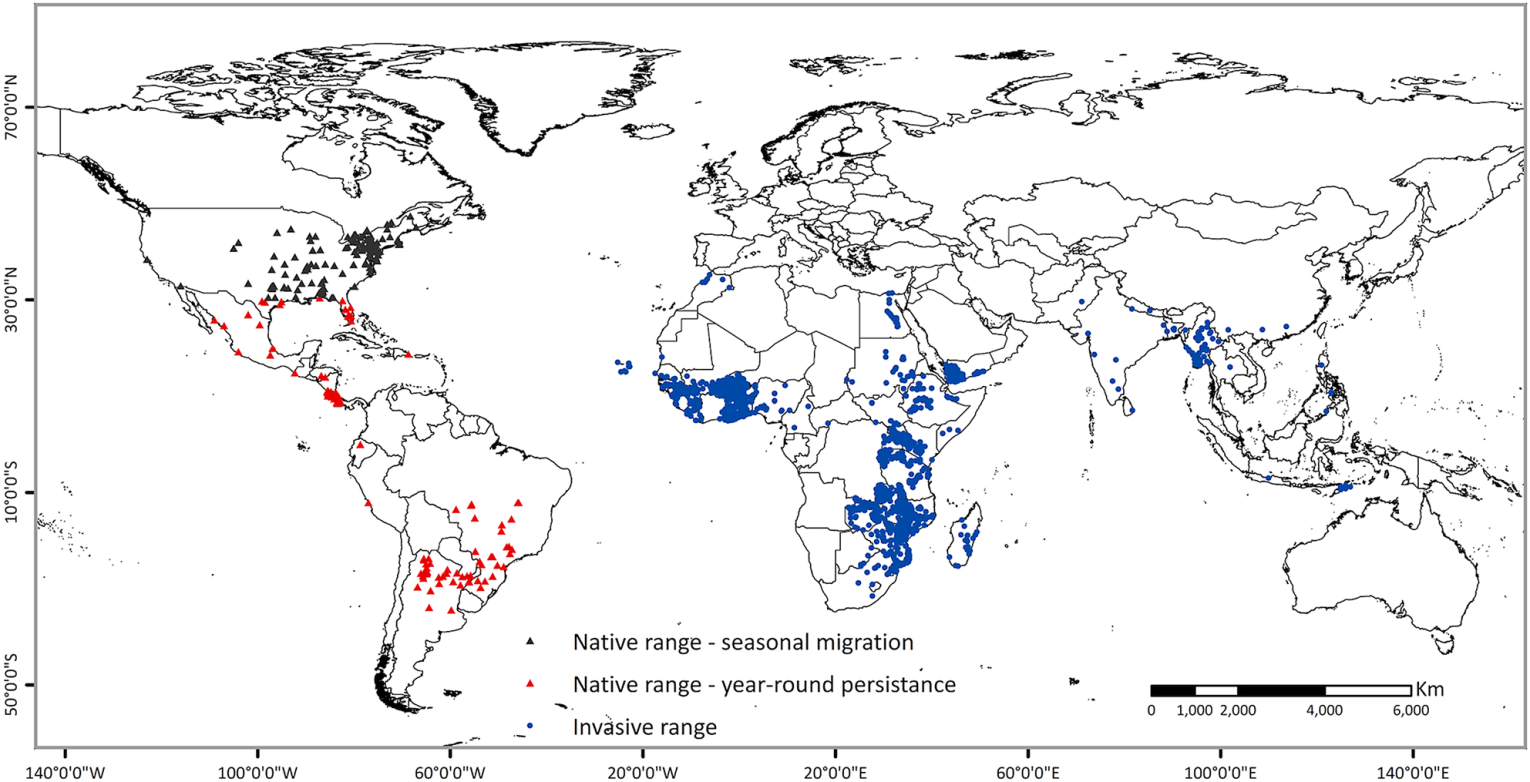
FAW presence confirmed locations in the world. Triangles represent FAW occurrence records from its native range—black triangles show areas that support seasonal population growth and red triangles show areas that support year-round population establishment. Blue circles show FAW occurrence records from its invasive range. ArcMap 10.8 (https://desktop.arcgis.com/en/arcmap/).

Overall, a total of 13,460 FAW global distribution records with either pheromone trapping (with confirmed FAW moth) or field scouting (with confirmed FAW larvae) data were collected. To make data visualization and manipulation easier, FAW occurrence records were spatially filtered to retain a single record in each 10-arc minute (~ 18 km) grid. This resulted in 2968 records (Africa—2591, Asia—150, North America—171 and South America—56) used for further analysis. The distribution records used in this study are shown in Fig. 1**.**

#### Climate data

The current suitability of FAW was modeled with the CliMond historical dataset interpolated at 10-arc minute (available at https://www.climond.org)^46^. This dataset consisted of long-term averages centered at the year 1975 for maximum and minimum temperatures, precipitation and relative humidity at 09:00 and 15:00 h. Future climatic suitability for FAW was projected using the 10-arc minutes gridded spatial resolution climate data for 2030, 2050, and 2080 retrieved from the CliMond (Version 2) in CLIMEX format^46^. The future climate projections used in this study were based on two global climate models (GCM), CSIRO-Mk3.0 GCM developed by CSIRO Atmospheric Research, Australia^47^ and MIROC-H GCM developed by Center for Climate Research, Japan. These were run with the A1B SRES (Special Report on Emission Scenarios) emission scenarios. The SRES A1B was chosen with the assumption that, in the future, the use of fossil intensive and non-fossil energy sources will be balanced. For an A1B emission scenario, the CSIRO-Mk3.0 and MIROCH-H GCMs predict a rise in temperature of 2.11 °C and 4.31 °C, respectively, by the end of twenty-first century^46,48^. Similarly, these two GCMs predict different rainfall patterns^49^.

The Intergovernmental Panel on Climate Change (IPCC) Fifth Assessment Report (AR5) released a new family of emission scenario called Representative Concentration Pathway (RCP), to replace the SRES family. This consists of four-climate change scenarios- RCP2.6, RCP4.5, RCP6 and RCP8.5. The closest similar RCP scenario to SRES A1B is RCP 6.0, which represents an intermediate emission scenario^50^. The temperature increase at the end of the twenty-first century for RCP6.0 scenario is projected to be 2.2 °C with a range of 1.4–3.1 °C, while for SRES A1B scenario, it is projected to be 2.8 °C with a range of 1.7–4.4^51^. Furthermore, the CO2 concentration by the end of the century for RCP6.0 is expected to reach 670 ppm, just below A1B (703 ppm)^50^.

#### Irrigation data

The information on global irrigation areas was derived from the Food and Agriculture Organization of the United Nations (http://www.fao.org/aquastat/en/geospatial-information/global-maps-irrigated-areas/).

#### Host crops data

Geographic distribution of maize and sorghum, two major host crops of FAW, were obtained from the EarthStat database (http://www.earthstat.org/) created by Monfreda et al*.*^52^.

### Model fitting

Using the “Compare Locations” modules in CLIMEX, the values of FAW parameters (Table 1) were determined (1) from previous studies’ findings on requirements for growth and development of FAW^53–56^, and (2) by fitting the projected distributions to the occurrence records of FAW in its native range and the recently invaded range in East Africa. We initially adopted parameter values from a previously conducted CLIMEX study on FAW^35,38^. Then, parameter values were determined through an iterative process to fit the simulated CLIMEX results to the known distribution of FAW in the world in 2020. FAW occurrence records in North America include reports of transient or migrant populations (Fig. 1, Supplementary Table S1). Therefore, during the model fitting process, we confirmed that the areas with migrant populations have a positive annual growth index (GI > 0) and an unsuitable eco-climatic index (i.e. EI = 0)^57,58^. The fitted values of CLIMEX parameters were then validated by comparing EI distribution of FAW with independent sets of FAW occurrence data collected in eight African countries (Burkina Faso, Ghana, Madagascar, Malawi, Mozambique, Liberia, Sudan, and Zambia), Asia, and Australia. The process of parameter fitting was repeated until the reliability and consistency of the model projections were established. The parameter values used in this study are listed in Table 1, and details are provided in the following sections.

#### Growth indices

##### Temperature index (TI)

The lower (DV1) and upper (DV2) optimal temperatures for FAW population growth were left unchanged at 25 °C and 30 °C, respectively^35^. These values are supported by multiple publications^53–56^. FAW reared at 25 °C constant temperature are less likely to emerge deformed^55^, and the adult moths have the highest adult longevity and fecundity^53^. A recent study by Du Plessis et al*.*^17^ identified 30 °C as the upper optimal temperature for FAW growth and development. The development rate of FAW increases linearly with increasing temperature from 18 to 30 °C, but declines when temperature increases above 30 °C^17^.

The limiting low temperature (DV0) was also kept the same at 12 °C^35,38^. FAW lacks any diapause mechanisms and overwinters only in warm and humid areas; hence, FAW cannot tolerate freezing temperature^14^. Wood et al*.*^59^ reported that a temperature above 10 °C is required for pupal eclosion. The pupae held at 10 °C live for 50–62 days but do not eclose^55,59^.

The limiting high temperature (DV3) was set at 36 °C because more than 50% of FAW reared at 35 °C or above exhibit physical deformity and die within 24 h of emergence^55^.

##### Moisture index (MI)

Not much is known about the relationship between soil moisture and the FAW lifecycle, so we adopted the species parameters from previous studies^35,38^, with some assumptions. The lower soil moisture threshold (SM0) was kept the same at 0.15 to allow FAW invasion in semi-arid areas in Africa. Silvain & Ti-A-Hing^60^ reported higher FAW populations (both adult moths and larvae) during rainy seasons than in the dry seasons. At any time, the larval population is affected by the amount of rainfall that was experienced three weeks earlier^60^. Although a heavy downpour reduces adult emergence by trapping moths in their pupation tunnel^61^, FAW larvae can tolerate substantial waterlogging conditions^35^. Therefore, the upper soil moisture threshold (SM3) was set to 2. Reducing the value of SM3 from 2.5 to 2 had no effect on defining the potential range of FAW. The lower (SM1) and upper (SM2) limits for optimal growth were left unchanged at 0.8 and 1.5, respectively^35^. The upper optimal soil moisture (SM2) value allows the persistence of FAW in tropical areas that experience high rainfall, such as Central America. Here, the SM value 0 indicates no soil moisture; SM 0.5 indicates soil moisture is 50% of soil water holding capacity, and SM > 1 indicates a run-off situation.

#### Stress indices (SI)

CLIMEX mainly uses four stress indices (SI: heat, cold, wet, and dry) to determine the species' geographical distribution. Species population growth occurs between the temperature parameters DV0 and DV3, and moisture parameters SM0 and SM3 (Table 1). Values outside of this range result in negative population growth.

##### Cold stress (CS)

The cold stress temperature threshold (TTCS) and cold stress accumulation rate (THCS) was decreased to 8 °C and − 0.005, respectively, to fit the FAW distribution in the Rio Grande valley Texas (overwintering site in North America), Mediterranean coast in North Africa and the Yunnan province (first FAW-invaded province) in China.

##### Heat stress (HS)

The heat stress temperature threshold (TTHS) for HS was kept the same at 39ºC to allow pest development in western African countries^35^. Heat stress accumulation rate (THHS) was set to 0.0025 week^−1^ to allow the pest development in Nile River basins in Egypt and irrigated areas in Yemen and Pakistan.

##### Dry stress (DS)

Dry stress indices were the same as those of the existing model of Du Plessis et al. ^35^. Soil moisture dry stress threshold (SMDS) was set to the same value as SM0, i.e., 0.15, and dry stress rate (HDS) was set to 0.005.

##### Wet stress (WS)

Soil moisture threshold for wet stress (SMWS) was set at the same value as SM3 in our model. The wet stress accumulation rate (HWS) was increased to 0.01 week^−1^ to exclude extremely wet areas from being suitable. This change does not limit the FAW persistence in areas with known FAW distribution but reduces the modeled risk in extremely wet areas.

#### Effective degree-days (PDD)

In CLIMEX, the PDD parameter indicates the degree-day above the minimum base temperature (DV0) necessary for species to complete one generation. Hogg et al*.*^62^ estimated PDD for FAW at 346.2 degree-days with base temperature of 13.8 °C while Du Plessis et al*.*^17^ calculated PDD value of 390 degree-days with base temperature 12.57 °C. In the current study, the base temperature was set at 12 °C. To get the same number of generations per year, PDD value was increased to 400-degree days.

### Irrigation

FAW was reported in dry areas in North Africa, Pakistan and Yemen. These areas did not fall within climatically suitable areas projected under rainfed conditions. These dry area records might reflect FAW populations able to persist only when irrigation is applied to sustain the crop. To simulate the effect of irrigation in FAW distribution, two irrigation scenarios were taken into account. First, the CLIMEX model for FAW was run using 2.5 mm day^−1^ as top-up irrigation throughout the year (Irrigation scenario I) to capture the risk posed by FAW in areas where cropping should be sustained by irrigation^35,42,57^. This top-up irrigation was added only when the weekly rainfall was less than 25 mm. Second, a composite FAW-risk map (Irrigation scenario II) was developed by combining the rainfed and irrigation scenario I results; EI from irrigation scenario I was mapped in areas under irrigation reported by Siebert et al*.*^63^ and EI for rainfed scenario was mapped elsewhere.

### Model performance

FAW occurrence records were overlaid on the projected layer surfaces to evaluate the model performance. EI values of the pixel where each FAW occurrence records lie were extracted from the projected raster layers. A histogram and a normal distribution curve were fitted on the projected EI values of each dataset under the current and projected future climates. Descriptive statistics were also generated from the extracted EI values to recognize the projected models that captured the presence records better. This analysis was used to measure and confirm the ability of the developed model to predict the FAW habitat areas successfully.

### Parameter sensitivity and model uncertainty analysis

CLIMEX model is a robust tool to assess the risk of pest invasion and establishment, but it includes several sources of uncertainty that need to be communicated to risk assessors and decision makers. The CLIMEX Version 4 has the parameter sensitivity and model uncertainty analysis tool available to evaluate the model. The sensitivity analysis identifies the degree to which each species parameter affects the projected areas of climatic suitability, whereas uncertainty analysis reflects the ability of the model to accurately predict the climatic suitability for a species. For the parameter sensitivity and uncertainty analysis, the default model parameters were run using the historic climate (CM10 1975H V1.2) under the rainfed scenario. Both parameter sensitivity and model uncertainty analysis were run for the entire world.

### Potential overlap between FAW and its major host maize

The projected suitability areas for FAW was overlaid on the projected distribution of its major host maize, to assess the potential co-occurrence of FAW and maize under the current and future climates. We performed the CLIMEX suitability analysis for maize using the maize-CLIMEX parameters from Ramirez-Cabral et al*.*^43^ (Supplementary Table S2). The maize-CLIMEX model developed by Ramirez-Cabral et al*.*^43^ did not include irrigation. In the current study, the maize-CLIMEX model was updated to include irrigation. Similar to FAW projections, future climatic suitability for maize distribution was projected using the 10-arc minute gridded spatial resolution climate data for 2030, 2050, and 2080, assuming A1B emission scenario. EI maps for maize (Supplementary Fig. S1) were created considering Irrigation scenario-II (i.e., EI values from the maize-CLIMEX model with irrigation was used in the irrigated areas and EI values from the maize-CLIMEX model without irrigation was applied elsewhere). Areas with EI value above 10 (i.e., suitable and optimal categories) were used to calculate the potential area overlap between FAW and maize distributions. Areas with EI value less than 10 (i.e., unsuitable and marginal categories) do not support or marginally support the species distribution. Therefore, those were excluded from the analysis to increase the comparability between the pest and its host maize.

We considered only maize for the pest-host overlap analysis because CLIMEX suitability analysis for maize is already published^43^. No such analysis is available for other host crops.

## Results

### FAW global potential geographical distribution under the current climate

**Figure 2:**
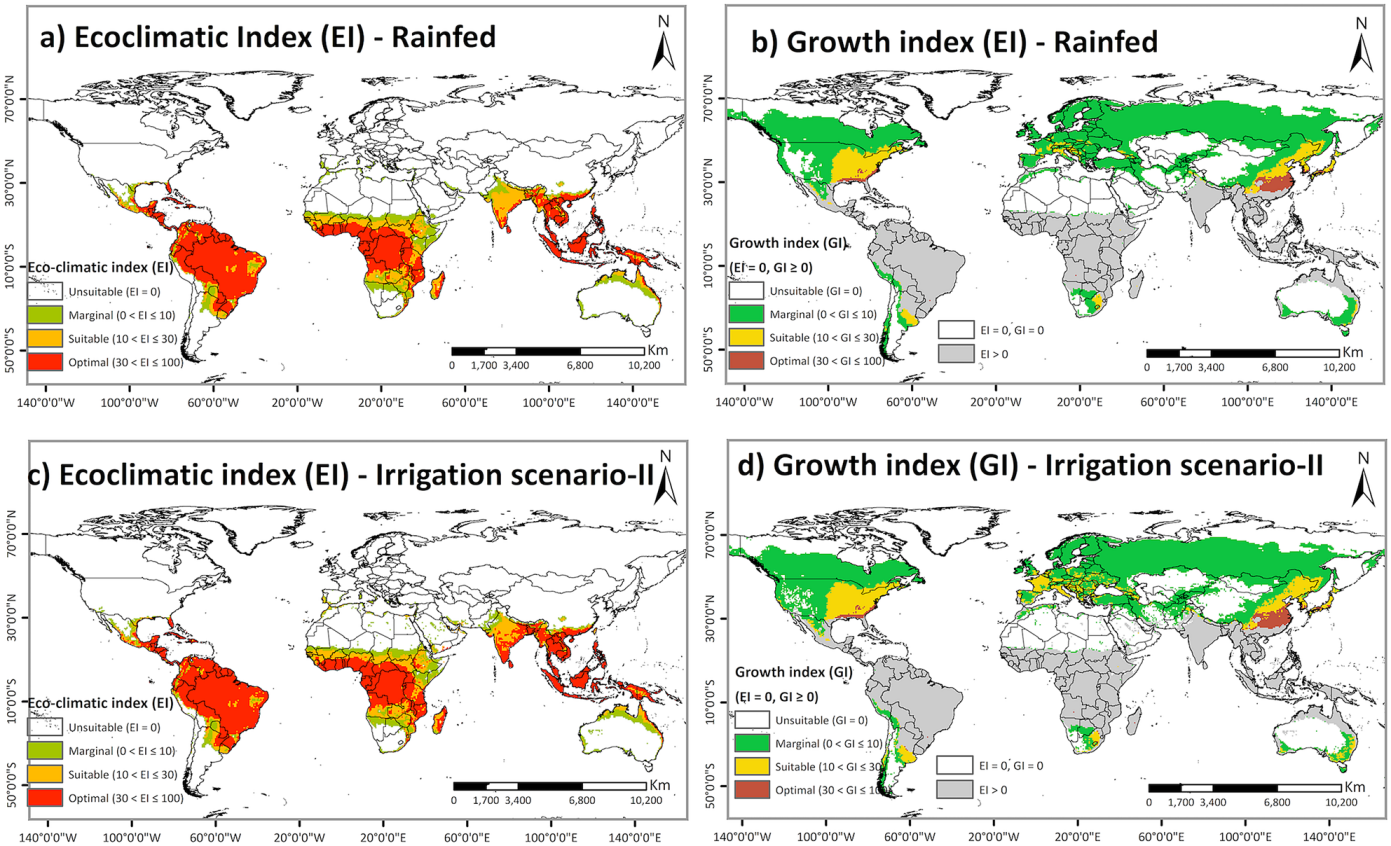
The projected global climate suitability for FAW population establishment and seasonal population growth under historic climate using CLIMEX. (**a**) Projected areas for year-round population establishment under rainfed condition. (**b**) Growth index (GI) for seasonal population growth under rainfed condition. (**c**) Projected areas for year-round population establishment under irrigation scenario II. (**d**) Growth index (GI) for seasonal population growth under irrigation scenario II. Areas with EI > 0 support FAW year-round population establishment, areas with EI = 0 but GI > 0 support FAW seasonal population growth and areas with EI = 0 and GI = 0 are unsuitable for FAW survival. ArcMap 10.8 (https://desktop.arcgis.com/en/arcmap/).

The potential projected global geographical distribution of FAW, ignoring the distribution of crop hosts and non-climatic barriers, corresponded well with the known present distribution of this species. The result shows that most of the world's tropical and subtropical climates are climatically suitable for year-round FAW establishment (with EI > 30) (Fig. 2a). This area increases substantially when looking at seasonal FAW population growth (with GI > 0): our model predicts that areas with humid-continental and Mediterranean climates have the potential to support FAW population growth during sometime of the year (Fig. 2b).

The model projected a notable difference in the potential distribution of FAW between irrigation scenario-II and the rainfed scenario, especially in North Africa, the Middle East and Australia (Fig. 2c,d). Many parts of North Africa and the Middle East become climatically suitable for FAW population establishment, when irrigation is available in these areas.

#### Native range

Potential FAW distribution in the Americas, its native range, is consistent with its known geographical range. The model confirmed that FAW over-wintering locations in the Americas are climatically suitable (with EI > 30) for the persistence of the FAW populations (Fig. 2a,c) under both rainfed condition and irrigation scenario-II. Furthermore, our model projected the expansion of the FAW geographical range to the eastern parts of North America with humid-continental and humid-subtropical climates during favorable seasons (with GI > 0, EI = 0) (Fig. 2b,d). In South America, the entire continent, except the Andes region and Argentina, has an optimal climate for FAW establishments.

#### Non-native range

**Figure 3:**
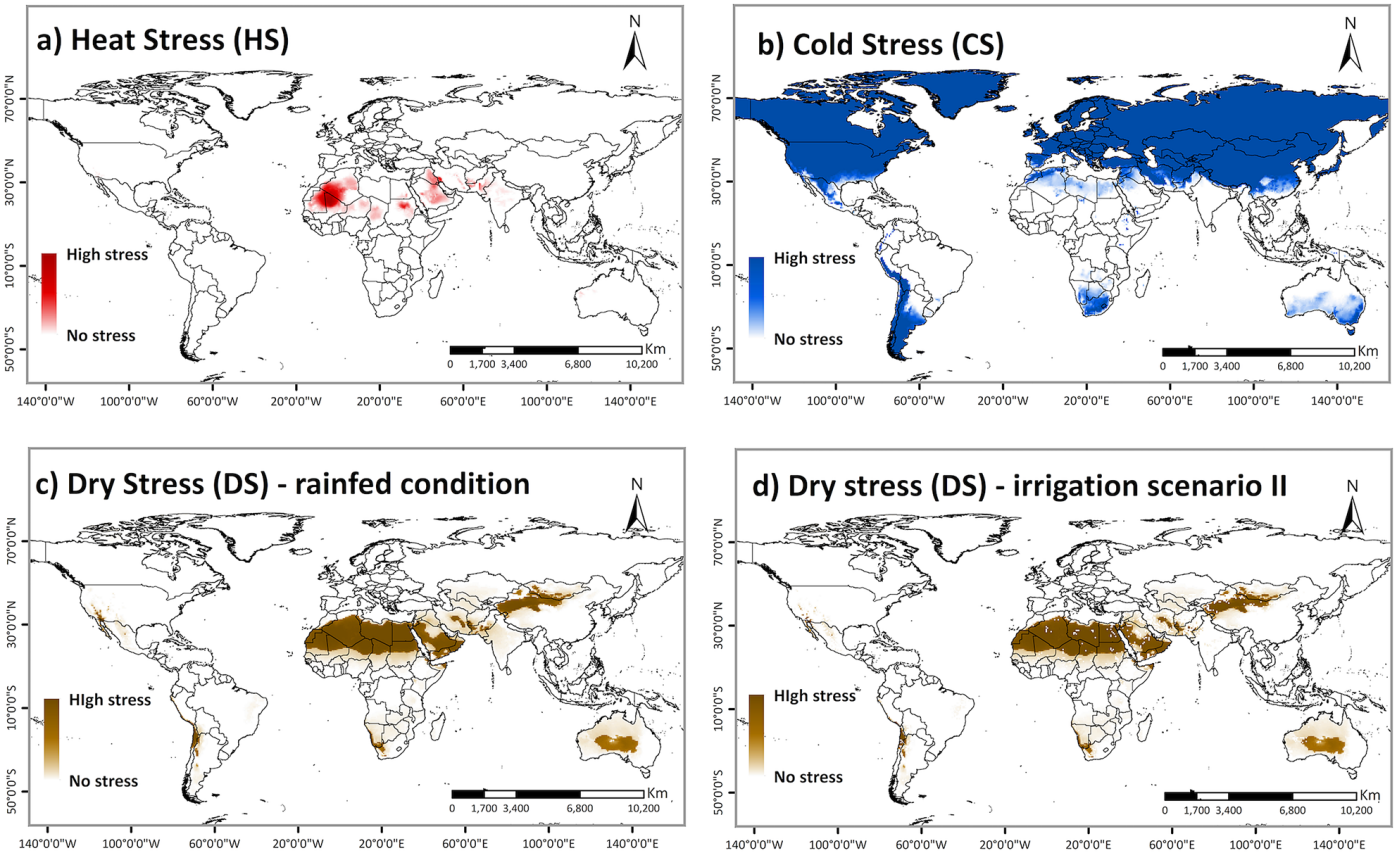
Abiotic stresses that limit FAW distribution and year-round establishment in the world. Areas with projected annual (a) heat stress, (**b**) cold stress, (**c**) dry stress (with rainfed condition), and (**d**) dry stress (with irrigation scenario II) that limit FAW population establishment under current climates. ArcMap 10.8 (https://desktop.arcgis.com/en/arcmap/).

Almost all areas in Africa, the non-native range, with known presence records were projected to be climatically suitable (with EI > 30) for FAW population persistence under rainfed conditions (Fig. 2a). A few occurrence records in North Africa did not fall into the region projected as suitable by the model. However, irrigation is used to sustain agriculture in those areas^63^ (Supplementary Fig. S2). Most of these locations become suitable when the irrigation scenario II is included in the model (Fig. 2c). About 27% of Africa's total area is projected to have an optimal climate for FAW population establishment, under irrigation scenario II (Fig. 2c). In eastern and central Africa, all countries except Somalia, Eritrea and Chad were projected to have suitable to optimal climatic for FAW population establishment and are under high risk. In western Africa, Guinea, Sierra Leone, Liberia, Côte d'Ivoire, Ghana, Togo, Benin, Nigeria, Sao Tome and Principe were also projected to potentially support year-round FAW populations. North African countries with tropical and subtropical desert climates were projected to be most unsuitable, with only irrigated areas and areas along the Mediterranean coast projected to have marginally suitable climates for FAW persistence. Heat and dry stresses would limit population establishment and growth in North African countries under the current climate (Fig. 3). Similarly, the Indian Ocean coast in South Africa is the only area in southern Africa that has an optimal environment for FAW persistence. Other parts in this region were not projected to support FAW population establishment due to dry and cold stress (Fig. 3b,d). However, availability of irrigation would render these dry areas suitable for FAW invasion and population growth (Supplementary Fig. S3).

Although Europe has an unsuitable climate for FAW year-round establishment (Fig. 2a,c), a few areas with humid-continental and Mediterranean climates are potentially suitable to support migrant FAW populations (with GI > 10) for a few generations per year (Fig. 2b,d). The risk of FAW invasion in Europe has become increasingly possible considering that FAW presence has recently been reported for Egypt and Mauritania. Furthermore, since extensive areas of host crop plants are readily available in southern Mediterranean Europe (Supplementary Fig. S4), these areas have potential to support transient populations under the current climate. Small regions of southern Italy, Spain, and Portugal have suitable to marginal climates for FAW year-round establishment, and these areas could be expected to be invaded first, as FAW has already reached the Nile Valley in Sudan, Egypt, and Mauritania. Yet, low winter temperatures in the majority of Europe would limit population growth and persistent establishment under the current climate (Fig. 3b).

FAW invasion has been confirmed in many countries in Asia in early 2018^20–23^. There is a considerable chance that FAW will extend beyond its current range in Asia. Based on historical climate data, South East Asian and South Asian countries have optimal climate conditions for FAW population establishment (Fig. 2a,c). In addition, areas in southeast China with a humid subtropical climate are also optimal for FAW population establishment. When weather is favorable, FAW could migrate seasonally to maize-growing areas with unsuitable or marginal climates for seasonal population establishment (Fig. 2b,d).

A large part of Australia is unsuitable for FAW persistence because it has a tropical and subtropical desert climate. However, areas with tropical wet and dry climate are projected to range from suitable to optimal category for FAW persistence (Fig. 2a,c), and regions with humid-subtropical climate are suitable and have the potential to support migrant FAW populations (Fig. 2b,d).

### FAW projection under future climate conditions

**Figure 4:**
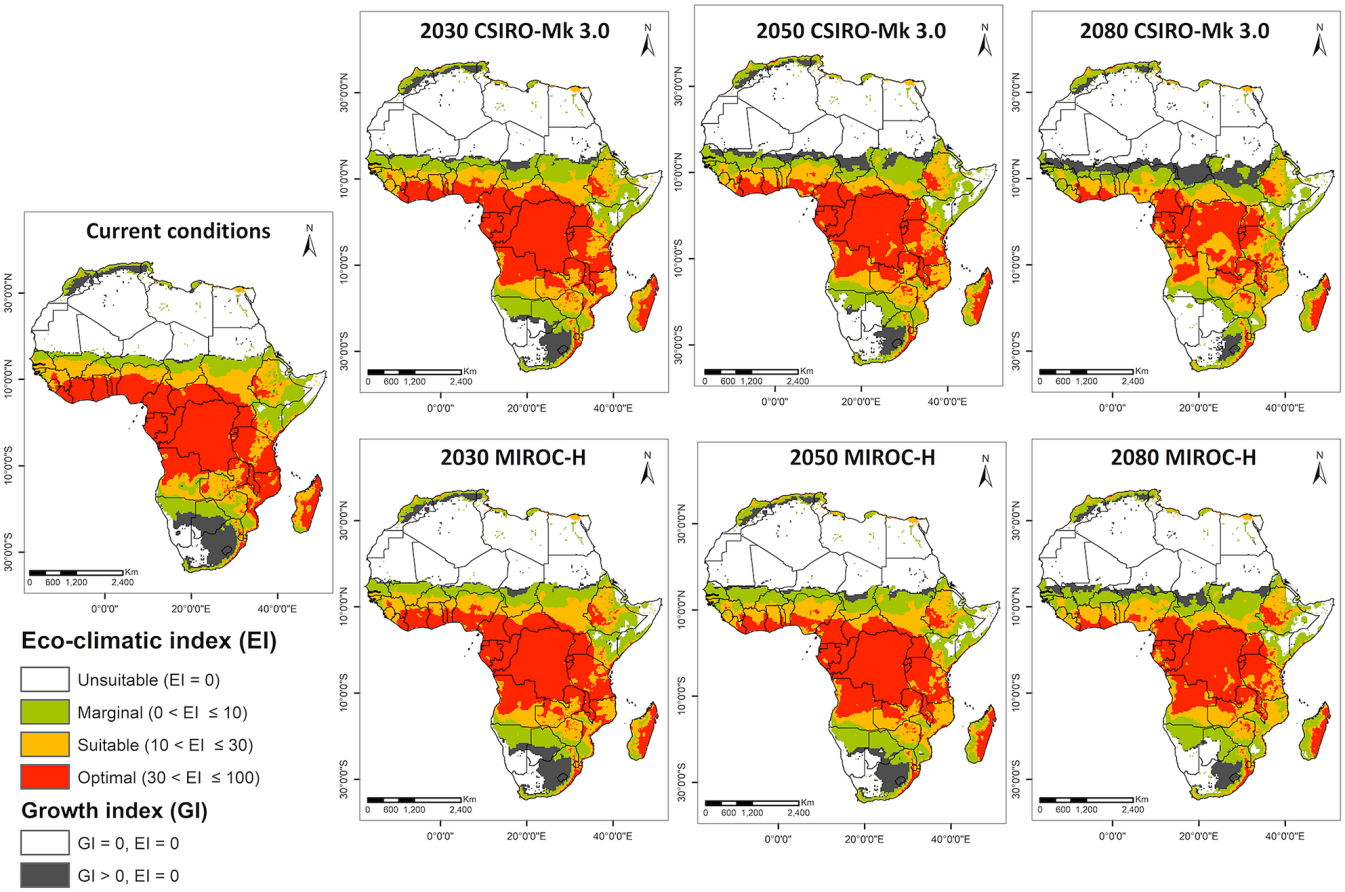
The climatic suitability areas for FAW population establishment and seasonal population growth, considering irrigation scenario II. These projections were based on the current and projected future climates (2030, 2050 and 2080) under CSIRO-Mk3.0 (top) and MIROC-H (bottom) GCMs. Areas with EI > 0 support FAW year-round population establishment, areas with EI = 0 but GI > 0 support FAW seasonal population growth and areas with EI = 0 and GI = 0 are unsuitable for FAW survival. ArcMap 10.8 (https://desktop.arcgis.com/en/arcmap/).

The projected FAW distributions in Africa under CSIRO-Mk3.0 and MIROC-H GCMs using the A1B scenario for 2030, 2050, and 2080 are shown in Fig. 4. Here, we considered irrigation scenario-II to project the areas suitable for FAW year-round persistence. The suitability projections under rainfed condition and irrigation scenario-I are included in the Supplementary Figs. S5 and S6. The future scenarios considering irrigation scenario-II project the gradual loss of climatically suitable areas for FAW population establishment and persistence in Africa. Although both GCMs project similar trends, the reduction in climatic suitability was more widespread under CSIRO-Mk3.0 than under MIROC-H GCM (Table 2, Fig. 4, and Supplementary Fig. S7).

**Table 2:** Areas subject to projected eco-climate suitability for FAW (*Spodoptera frugiperda*) persistence under current and future projected climates, considering irrigation scenario II.

	EI under current climate scenario		Percentage change in areas under future projected climate from the current climate					
	Total area (10^6^ km^2^)	Percentage	CSIRO-Mk3.0			MIROC-H		
			2030	2050	2080	2030	2050	2080
Optimal	8.9	26.8	− 2.4	− 5.5	− 11.6	− 1.8	− 3.7	− 7.2
Suitable	5.5	16.4	− 0.8	− 1	0.3	0.2	− 0.2	− 0.8
Marginal	4.7	14	1.7	2.2	1.3	1	2.3	3.7
Unsuitable	14.3	42.8	1.5	4.4	10	0.6	1.6	4.3

The percentage values for future projected climate are the percentage change in these areas under future projected climate from the current climate.

**Figure 5:**
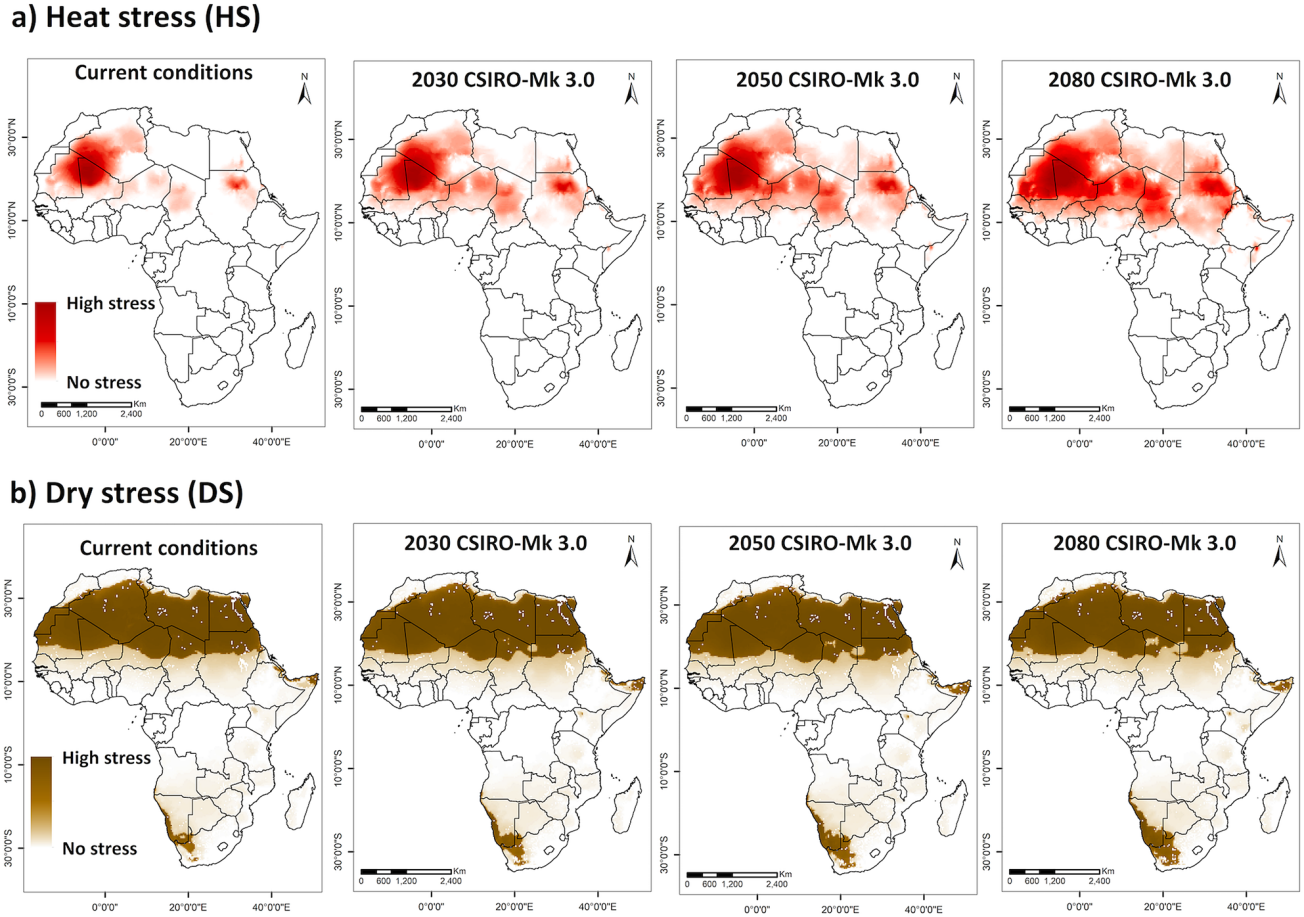
Abiotic stresses that limit FAW distribution and year-round establishment in Africa. Projected annual (**a**) heat stress and (**b**) dry stress for FAW under current and projected future climates (2030, 2050 and 2080) using CIRSO-Mk3.0 GCM, considering irrigation scenario II. ArcMap 10.8 (https://desktop.arcgis.com/en/arcmap/).

For 2030, both CSIRO-Mk3.0 and MIROC-H GCMs projected a reduction of approximately 2% in areas with optimal climatic suitability, with a shift from optimal to suitable or marginal climate. All currently suitable countries in West Africa were projected to gradually retract from the optimal range for FAW by 2030, and many countries lose it by 2080 due to heat and dry stress (Figs. 4 and 5). Areas of optimal suitability are also projected to decrease in countries, such as South Sudan and Mozambique. For the remaining countries with current climatic suitability, the projected changes in climate for the next 10 years would not affect their FAW suitability. Some areas in Angola, Zambia, Zimbabwe, and Tanzania currently classified as suitable category are projected to improve to optimal in the warmer future climates.

The modeled projections of climatic suitability for 2050 under the two GCMs indicated slight differences. The projected decrease in area with optimal suitability was higher under the CSIRO-Mk model (5.5%) than projections under the MIROC-H model (3.7%). While all the currently suitable countries (except Angola, Zambia, and Zimbabwe) lose climatic suitability, the reduction is significant in Eastern and West African countries (Supplementary Fig. S7). There would be no or very little change in the suitability of areas in Central African countries, such as Cameroon, Equatorial Guinea, Gabon, Congo, and the Democratic Republic of Congo. In Eastern Africa, Uganda, Rwanda, and Burundi are the only countries with optimally suitable climates throughout the countries by 2050 under both GCMs.

The projections under both GCMs show a reduction in climatic suitability, changing from optimal to marginal by 2080, for African countries south of the Sahara and north of the Kalahari Desert. The CSIRO-Mk3.0 model projected larger decreases in areas of optimal suitability, with a 10% reduction, compared with only a 4% reduction under MIROC-H. Under both models, nine countries—Senegal, Mali, Niger, Sierra Leone, Burkina Faso, Chad, Togo, Benin and South Sudan—showing currently optimal to suitable climate, will no longer support or only marginally support FAW year-round persistence by 2080. The region where the suitability would be least affected by projected climate changes under the MIROC-H model is central Africa, except Chad and the Central African Republic.

Under the climate change scenario, the current potential range of FAW is expected to decrease mainly due to heat and dry stress in Africa (Fig. 5). Hot and dry summers will create stressful conditions that prevent FAW from completing its life cycle and persisting year-round in areas that are now suitable for year-round persistence. However, there is still a chance that FAW escapes the heat and dry stress during hot summers by migrating to Central and Eastern Africa and reinvading the marginal or unsuitable areas during the warm winter season each year.

Those African countries that lie along the equator will likely support the long-term persistence of FAW populations and experience a stable risk throughout the year (Fig. 4). These areas include parts of Ethiopia, Kenya, Uganda, Rwanda, Burundi, Tanzania, Zimbabwe, Zambia, Angola, Democratic Republic of Congo, Republic of Congo, Gabon, Equatorial Guinea, and Madagascar. These areas could also serve as FAW hot spots from where FAW migrate each year to re-infest a succession of cropping areas in other regions with marginal and suitable growth index (GI > 0) but unsuitable eco-climatic index (i.e. EI = 0) (Fig. 4).

### Potential overlap between FAW and its major host plant maize

Under the current climate conditions, 10.1 million km^2^ (i.e., 33%) of the land area in Africa has suitable to optimal climate for both maize and FAW (Table 3). Under the projected future climates, the area with co-occurrence potential would decrease over time (Table 3, Supplementary Fig. S8). However, by the end of 2080 under CSIRO-Mk3.0 and MIROC-H GCMs, 13% to 16% of the area in Africa, respectively, would still support both FAW and its host plant maize. These areas of co-occurrence would have high potential to serve as a breeding spot or hot spot, like the southern part of Florida and Texas in the USA^28^.

**Table 3:** Total land area where FAW and its major host crop maize can potentially co-occur under the current and projected future climates in Africa.

Climate change Scenario	Area (sq. km)
Current climate	10,067,030
CSIRO 2030	8,159,690
CSIRO 2050	6,130,125
CSIRO 2080	3,878,571
MIROCH 2030	8,626,420
MIROCH 2050	7,224,368
MIROCH 2080	4,845,937

### Parameter sensitivity and performance analysis

The modeled potential distribution of FAW using the recent CLIMEX model is highly sensitive to change in the cold stress temperature threshold (TTCS) and limiting low moisture (SM0), with 1 and 0.8% impact, respectively (Supplementary Table S3). Other parameters have less than 0.5% sensitivity to the projected potential range, suggesting that TTCS and SM0 were the fundamental parameters for fitting the model simulation into the actual distribution.

**Figure 6:**
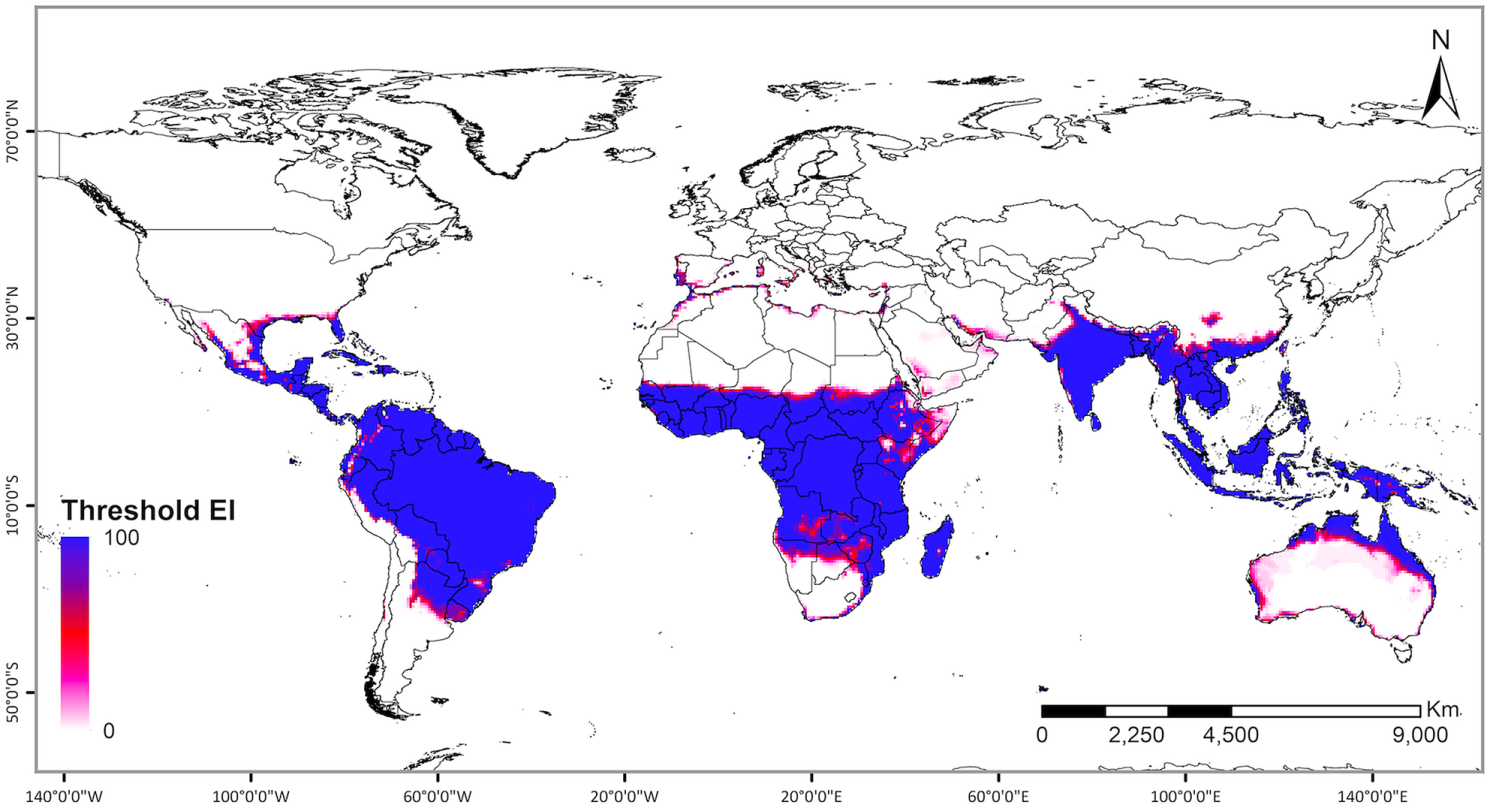
CLIMEX model uncertainty analysis. The proportional model agreement (%) for sampled parameter uncertainty. ArcMap 10.8 (https://desktop.arcgis.com/en/arcmap/).

The model uncertainty analysis indicates that there is a greater degree of geographical uncertainty about the ability of FAW to persist in drier areas (e.g. Sub-saharan Africa, Australia and the Middle East) than colder areas (e.g. USA and Mexico, Southern China) (Fig. 6).

The high proportion (0.98) of the occurrence records in the validation area falls within the area of projected suitable to optimal categories for FAW establishment (Supplementary Table S4), thereby reflecting the reliability, accuracy and robustness of the model. The CLIMEX model under current climate had an EI mean of 38.8 and EI median of 37.1, suggesting that a considerable proportion of the extracted EI values were within the optimal suitability threshold of 30–100 (Supplementary Fig. S9). The model under the current conditions had the highest EI mean of 38.8 and EI median of 37.1, while the CSIRO 2080 model had the lowest EI mean of 30.9 and EI median of 31.8. This indicates that the accuracy of models reduces with future projections. This implies that there is a possibility of a niche range shift of FAW based on the climate suitability in future.

## Discussion

The global geographical distribution of FAW projected with the CLIMEX model closely matched the current distribution of FAW. The model set out to predict areas where FAW populations could establish themselves permanently; that is, their presence is year-round with multiple generations. Based on the CLIMEX projections under present climate conditions, the world’s tropical and subtropical climates are suitable for the year-round establishment of FAW, whereas temperate climates are at risk of seasonal invasions. In Africa, a large part of east, west and central Africa (i.e., 57.2% of the total area) is currently suitable for FAW population establishment. In the future, areas of climatic suitability for FAW establishment are expected to gradually decrease over time mainly due to heat and dry stress. However, FAW may still survive and become established in significant areas in Africa (i.e., 47.2% and 53% of the total area under the CSIRO-Mk3.0 and MIROC-H GCMs, respectively) by the end of 2080. If certain conditions are met, these established persistent populations, could, in turn, serve as a source of seasonal invasions and migrate into less favorable climatic regions. Therefore, it is likely that FAW will establish permanent populations and cause substantial damage and economic losses to certain crops production every year in Africa unless FAW populations can be effectively managed.

Our CLIMEX model projects that, under the current climate, there is an increased risk of global FAW invasion and establishment. Outside of its native range in North and South America, the current climatic conditions in many parts of Africa, South and South-East Asia, southeastern parts of China, the north coast of Australia, and a few pockets in Europe are favorable for FAW invasion and establishment. Until 2016, FAW was confined to areas with a subtropical wet and dry climate in the Americas. After invading Africa in 2016, FAW has spread rapidly throughout vast regions of Africa that have a climate similar to its native range. By 2020, FAW has further spread to the Middle East, South Asia, South East Asia, and Australia^18,24^. While FAW has not yet invaded Europe, our model identifies several pockets in Europe that are climatically suitable for FAW invasion, suggesting that FAW has not reached its full potential range and its range is still expanding.

The model suggests that extreme temperatures, cold winters and hot summers, as well as limited soil moisture, are the most critical factors constraining the survival of FAW. Currently, cold stress limits FAW’s potential to permanently establish in northern North America, southern parts of South America, the northern part of Asia, and Europe. Presently, the permanent establishment of FAW in these regions is not possible because FAW does not diapause^14^. However, if global temperature increases as projected, some areas currently classified as climatically unsuitable may become suitable for FAW invasion and establishment in the future. Expansion towards the north has already been reported for several invasive insect species under climate change^64,65^.

Similarly, dry stress limits FAW’s establishment in North African countries, the Middle East, and a large part of Australia. In the future, these areas are projected to experience a reduction in total annual rainfall and would not be expected to support FAW populations. However, irrigated crop fields in these areas could support seasonal FAW populations during warm winters.

In Africa, central, eastern, and western African countries with sub-tropical wet and dry climates offer optimal conditions for FAW population establishment and are under high risk. These areas could serve as overwintering areas and sources of seasonal invasions to other parts of Africa, in the same way, that southern Florida, Texas, and Mexico serve in North America^28^. The projected FAW overwintering areas in the present study are consistent with those in previous studies^26,35^. Further, the projected climatic suitability for FAW also closely matches the projected distribution of maize, its preferred host under current and future climates (see Ramirez-Cabral et al*.*^43^ for global distribution maps of *Zea mays*), which further enhances the probability of FAW to invade and even establish persistent populations in the areas projected by our model.

In Africa, the northern part of FAW's current distribution is currently limited by extreme heat and dry stress. The permanent establishment of FAW in this region is unlikely, as FAW cannot tolerate extreme heat^53–55^. However, microclimates near maize growing areas in the Nile River basins and irrigated zones could provide channels of suitable habitat^66^ and support the establishment of FAW permanent populations. In addition, a small portion of the Mediterranean coast in North Africa is suitable for FAW persistence but has not yet been invaded. The arrival of FAW in those areas by its own means is unlikely because FAW has to migrate across the Sahara and face these harsh desert conditions. Yet, since FAW has already invaded the humid irrigated zones of Aswan Governorate in South Egypt^19^, we should not rule out the possibility of seasonal invasion of the north through natural migration. Migrant FAW moths could use maize fields along the Nile River basins as ‘stepping-stones’ before finding a suitable location along the North African coast. The flight biology of FAW in North America suggests that it can complete such a long-distance migration^28^. The establishment of significant populations along the North African coast and the Nile River basin will increase the threat to North Africa and Europe through seasonal migration^67^. The risk posed by migrant FAW populations north of the Sahara should not be ignored, since a similar scenario occurs in North America where the climate also does not support FAW establishment, yet crops are still devastated by seasonal invasions of FAW^28^.

Furthermore, several pest species have crossed unfavorable geographic barriers by ‘piggybacking’ on human transit routes. Therefore, intra-continental travel and trade increase the risk of FAW introduction to the North African coast and Europe^26,27^. Rwomushana et al*.*^4^ reported several interceptions of FAW in consignments from Africa. Therefore, to prevent travel-assisted introduction of the FAW in these places, close monitoring of the FAW invasion and activity in North Africa as well as adopting strict phytosanitary measures are and will continue to be necessary. In addition, pest species, including FAW, can move long distances aided by meteorological phenomena such as storm fronts^68^. While the present study did not consider it, an analysis of synoptic and mesoscale wind and storm patterns may be useful, especially since these patterns are projected to change over time^69,70^.

The climate around the globe is changing, and the distribution of areas climatically favorable to FAW persistence will change accordingly. Our results suggest that under future climate scenarios, the projected distribution of FAW in Africa will contract in both northern and southern regions towards the equator. These findings are in line with the results of a previous study, which also projected a decrease in climatically suitable areas in the Americas under the climate change scenario^38^. These range contraction projections are similar to those made for its preferred host, *Z. mays*^43^ (also see Supplementary Fig. S4). The projected decrease in suitable climate in the northern and southern range in Africa is due to a significant increase in heat and dry stress caused by increased temperatures. During hot and dry summers, FAW pupae do not generally hatch, and if they do, their ability to fly and search for mating partners is compromised^55,56^. Similarly, exposure to high temperatures for extended periods also significantly reduces fecundity and increases mortality in FAW^53^, leading to population decline in formerly suitable areas. However, these areas could be re-infested every year from migrant populations, thereby resulting in potential damage.

Our model projected a high risk of the permanent establishment of FAW in Africa. The year-round availability of host plants and warm and moist winters is optimal for FAW populations' long-term persistence in Africa. Although hot summer temperatures are likely to exclude the FAW from the warm habitat in humid-tropics in Africa, the overwintering populations in sub-tropical wet and dry climate areas could re-infest these areas every year during favorable seasons. Records of FAW occurrence in hotter and drier areas than those in its native regions suggest that FAW is extremely heat-tolerant, more so than we thought in the past. Since there are no detailed studies on FAW biology and ecology in Africa, we used temperature-dependent life history data from the studies conducted in the Americas during the 1960s–1990s to predict infestation areas with suitable climatic conditions. However, FAW may have adapted and become more heat tolerant than previously reported. Therefore, any change in the insect's ability to survive in extreme climate conditions (in this case, tolerance to dry and heat stress) would expand the projected range of distribution^71,72^. Hence, further studies are necessary to assess FAW's biological and ecological adaptation to the African continent using natural populations sourced from different climatic niches^73^.

The large area overlap between the projected geographical distributions of FAW and its major host maize also indicates the substantial ecological and economical risk posed by this species. Under current climate, 33% of the total land area in Africa has suitable to optimal climate for FAW persistence and maize cultivation. Since this species feeds on over 350 plant species, the area overlap between FAW and it’s host crops could be greater than 33%. This FAW-maize overlap area is expected to decrease over the next 50–60 years, mainly due to loss of climatic suitability for maize cultivation. FAW could still persist in areas outside the FAW-maize overlap areas if other host crops are present. Our analysis only shows the potential area overlap between FAW and host crop maize, assuming that maize is grown in all the areas climatically suitable for maize cultivation. Therefore, actual areas of maize and other host crops cultivation should be considered in the future studies.

The geographic distribution of FAW is determined by biotic interactions, abiotic factors, and its active or passive movement^74^. This study focuses on abiotic factors for temperature-dependent life history events to map climatically suitable areas for FAW because abiotic factors such as climatic variables are considered prime factors affecting species distribution at the continental and global scales^75^. Therefore, we examined the influence of climate change only on FAW's range of invasion and establishment. At these large scales, the results from the bioclimatic models alone are sufficient to suggest which regions are at high risk for pest invasion and establishment under future climates^75^.

Biotic factors should be incorporated in the bioclimatic model results to provide a more refined understanding of the species distribution under changing climates^74^. FAW requires a suitable place to live and complete its lifecycle over several generations. FAW also requires an adequate supply of host plants in synchrony with its lifecycle, without which it cannot persist in that environment. Furthermore, FAW's interactions with other species, such as natural enemies and competitors in a particular region, affect species distribution. Unfortunately, at this point combining all these factors in the bioclimatic model would increase the complexity and is not currently possible^74,75^.

The projected distribution of the FAW in Africa has indicated that FAW will severely impact agriculture over the next several decades. Insect pest management will be more challenging in a changing climate since climatic factors affect the timing of pest infestation, host preference, the efficacy of chemical and biological measures of control, and their utilization within integrated pest management strategies^76^. In this case, modeling habitat suitability for the pest under current and future climatic conditions provides robust tools and recommendations across multiple stakeholder levels and geographical scales. The current study will guide farmers, extension agents, researchers, policymakers, and public and private sectors to develop risk assessment protocols and climate-smart pest management strategies to prevent or reduce the economic loss due to FAW.

## Conclusion

The FAW poses a considerable threat to farmers worldwide. Projection of the pest distribution and its potential to establish in a targeted geographical location is crucial in enhancing preparedness, particularly selecting appropriate pest management control. Since FAW performance and survival is limited by temperature and humidity, we constructed a model that predicts areas suitable for FAW occurrence under current and future climate conditions. Here we considered two fundamentally different scenarios: areas where FAW persist year-round with multiple generations per year, and areas where FAW invade seasonally and each invasion starts from persistent populations. Under current climatic conditions, FAW has not yet reached all areas where it could potentially establish year-round populations, and therefore also not all areas that it can occasionally or seasonally invade. Under projected global temperature increases, the optimal areas for FAW persistence will shrink and, with them, the areas of seasonal invasions. Our model does not consider the multitudes of biotic interactions. However, it appears that at least the distribution of maize, FAW’s preferred host plant, follows a similar pattern, which means that under future climatic conditions successful cultivation of maize will require successful management of FAW populations.

## Supplementary Information

Supplementary Information 1.

Supplementary Information 2.
