# Supplementary material for: Potential distribution of fall armyworm in Africa and beyond, considering climate change and irrigation patterns
Source: Sci Rep. 2022 Jan 11;12:539. doi: 10.1038/s41598-021-04369-3 (PMC8752590; doi:10.1038/s41598-021-04369-3)
Supplement: Supplementary file 1 — Supplementary Information 1. [file 41598_2021_4369_MOESM1_ESM.docx]

**Potential distribution of fall armyworm in Africa and beyond, considering climate change and irrigation patterns**

**Supplementary materials:**

Bipana Paudel Tamilsena^1^, Saliou Niassy^2^, Emily Kimathi^2^, Elfatih. M. Abdel-Rahman^2^, Irmgard Seidl-Adams^1^, Mark Wamalwa^2^, Henri E. Z. Tonnang^2^, Sunday Ekesi^2^, David P. Hughes^1,3^, Edwin G. Rajotte^1^, Sevgan Subramanian^2^

**The following supporting information is available for this article:**

**Supplementary Fig. S1.** The area with potential climate suitability for maize distribution under the current and predicted future climates (2030, 2050 and 2080), considering irrigation scenario II. Maize-CLIMEX parameters were adapted from Ramirez-Cabral *et al*.^43^ ArcMap 10.8 (https://desktop.arcgis.com/en/arcmap/).

**Supplementary Fig. S2. Irrigated areas in the world.** This map is based on irrigated areas data on obtained from *Siebert et al.*^17^. ArcMap 10.8 (https://desktop.arcgis.com/en/arcmap/).

**Supplementary Fig. S3. The projected global climate suitability for FAW population establishment and seasonal population growth under historic climate using CLIMEX.** (a) Projected areas for year-round population establishment under irrigation-I scenario. (b) Growth index (GI) for seasonal population growth under irrigation-I scenario. Areas with EI > 0 support FAW year-round population establishment, areas with EI = 0 but GI > 0 support FAW seasonal population growth and areas with EI = 0 and GI = 0 are unsuitable for FAW survival. ArcMap 10.8 (https://desktop.arcgis.com/en/arcmap/).

**Supplementary Fig. S4. Global distribution of maize and sorghum, two major host crops of FAW.** The map is based crop data obtained from the EarthStat database (<http://www.earthstat.org/>) created by Monfreda *et al.*^52^ ArcMap 10.8 (https://desktop.arcgis.com/en/arcmap/).

**Supplementary Fig. S5.** **The climatic suitability areas for FAW population establishment and seasonal population growth, considering rainfed conditions.** These projections were based on the current and projected future climates (2030, 2050 and 2080) under CSIRO-Mk3.0 (top) and MIROC-H (bottom) GCMs. Areas with EI > 0 support FAW year-round population establishment, areas with EI = 0 but GI > 0 support FAW seasonal population growth and areas with EI = 0 and GI = 0 are unsuitable for FAW survival. ArcMap 10.8 (https://desktop.arcgis.com/en/arcmap/).

**Supplementary Fig. S6.** **The climatic suitability areas for FAW population establishment and seasonal population growth, considering irrigation scenario-I.** These projections were based on the current and projected future climates (2030, 2050 and 2080) under CSIRO-Mk3.0 (top) and MIROC-H (bottom) GCMs. Areas with EI > 0 support FAW year-round population establishment, areas with EI = 0 but GI > 0 support FAW seasonal population growth and areas with EI = 0 and GI = 0 are unsuitable for FAW survival. ArcMap 10.8 (https://desktop.arcgis.com/en/arcmap/).

**Supplementary Fig. S7. Impact of climate change on climatic suitability area (EI) for FAW population persistence.** White indicates no change, red indicates an increase, and blue indicates decrease in EI values under the future projected climate from the current climate. Depth of color indicates the degree of changes in EI value. ArcMap 10.8 (https://desktop.arcgis.com/en/arcmap/).

**Supplementary Fig. S8.** Potential overlap between FAW and maize, major host of FAW, under the current and projected future climates considering irrigation scenario-II. Red indicates areas with potential overlap between the pest and its host crop. ArcMap 10.8 (https://desktop.arcgis.com/en/arcmap/).

**Supplementary Fig. S9.**  **Statistical analysis to evaluate the CLIMEX model performance.**

**Supplementary Table 1.** FAW occurrence records from the Americas, the native range of FAW.

**Supplementary Table S2:** CLIMEX parameter values used for modeling the distribution of maize (*Zea mays*).

**Supplementary Table 3.** CLIMEX parameter sensitivity values for *Spodoptera frugiperda* (FAW) parameters listed in Table 1, as applied to the CM10 1975H V1.2 global dataset under rainfed conditions.

**Supplementary Table 4:** Number of FAW occurrence records in Africa that fall into each categories of eco-climatic index under rainfed conditions and irrigation scenario-II.


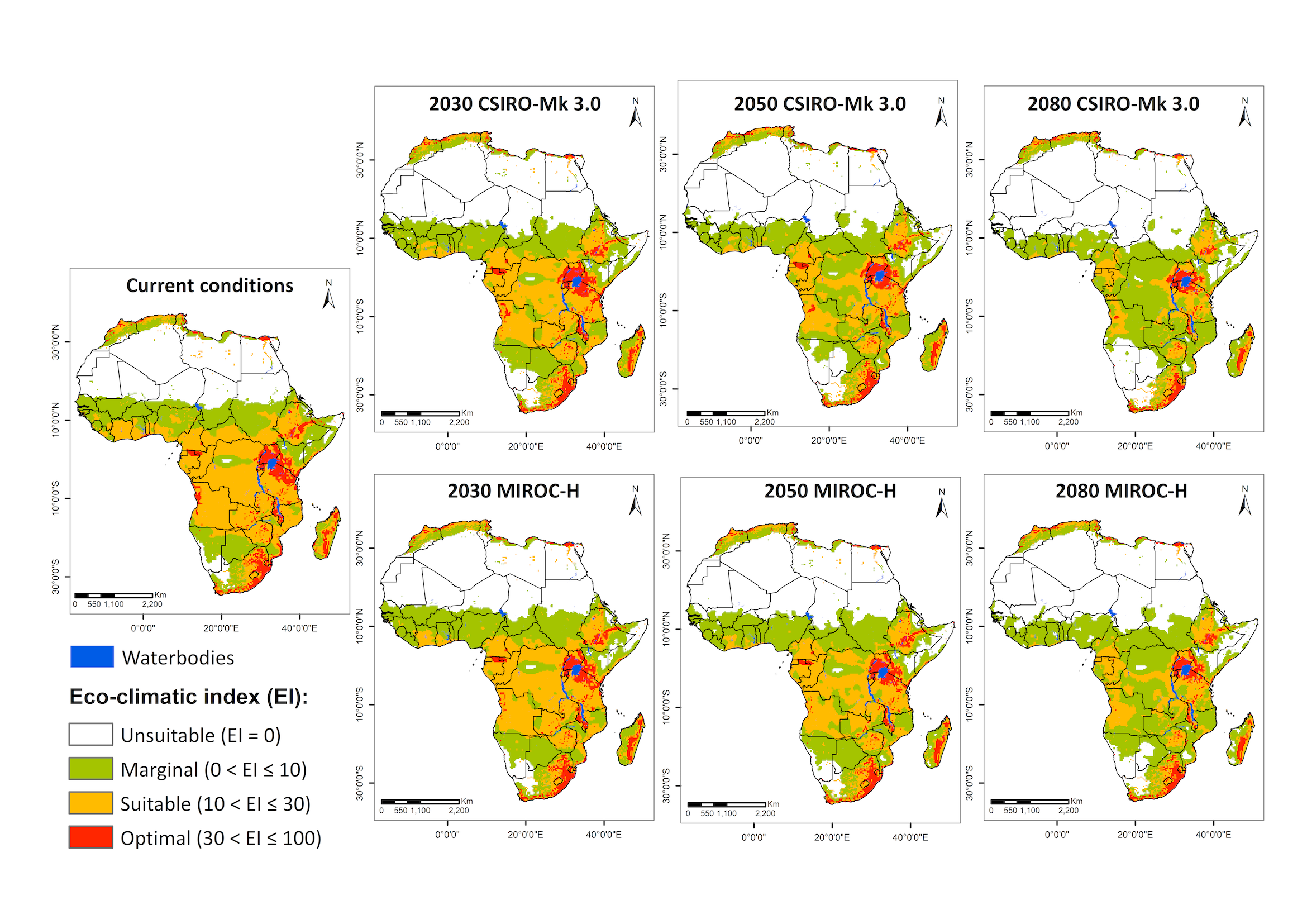


**Supplementary Fig. S1.** The area with potential climate suitability for maize distribution under the current and predicted future climates (2030, 2050 and 2080), considering irrigation scenario II. Maize-CLIMEX parameters were adapted from Ramirez-Cabral *et al*.^43^ ArcMap 10.8 (https://desktop.arcgis.com/en/arcmap/).


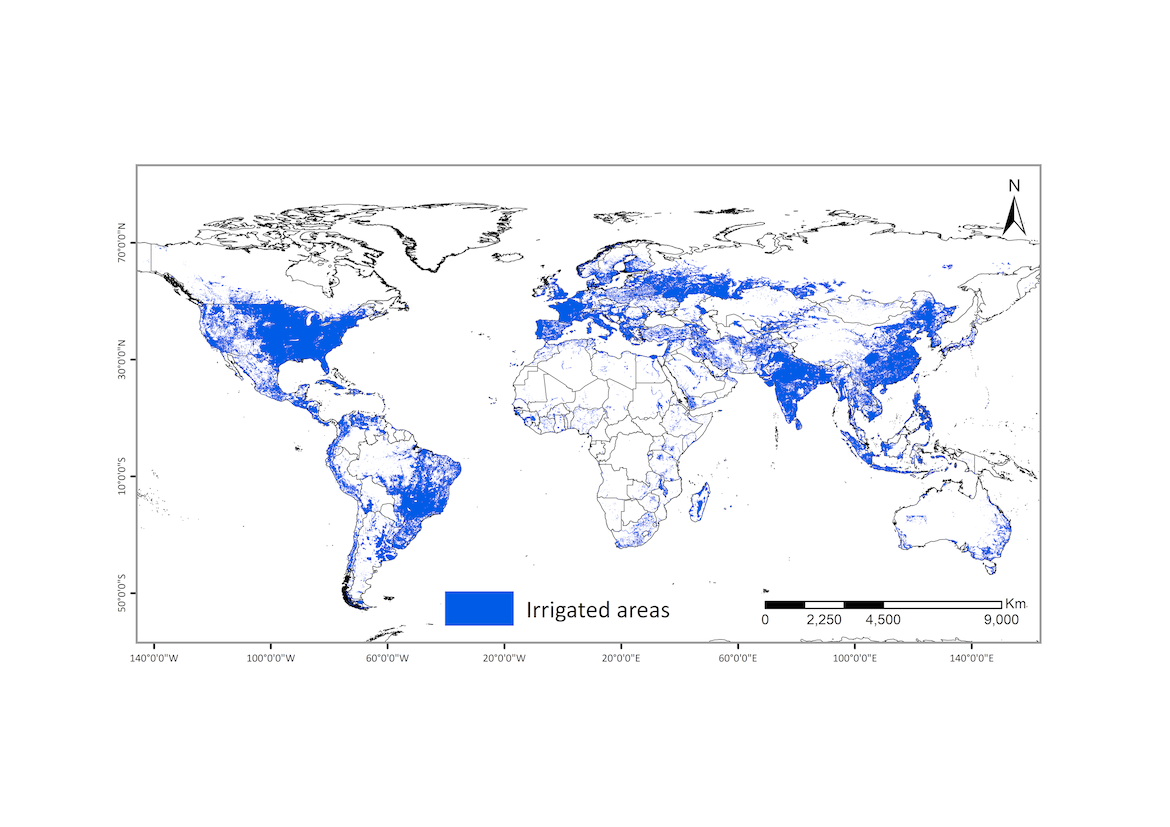


**Supplementary Fig. S2. Irrigated areas in the world.** This map is based on irrigated areas data on obtained from *Siebert et al.*^17^. ArcMap 10.8 (https://desktop.arcgis.com/en/arcmap/).


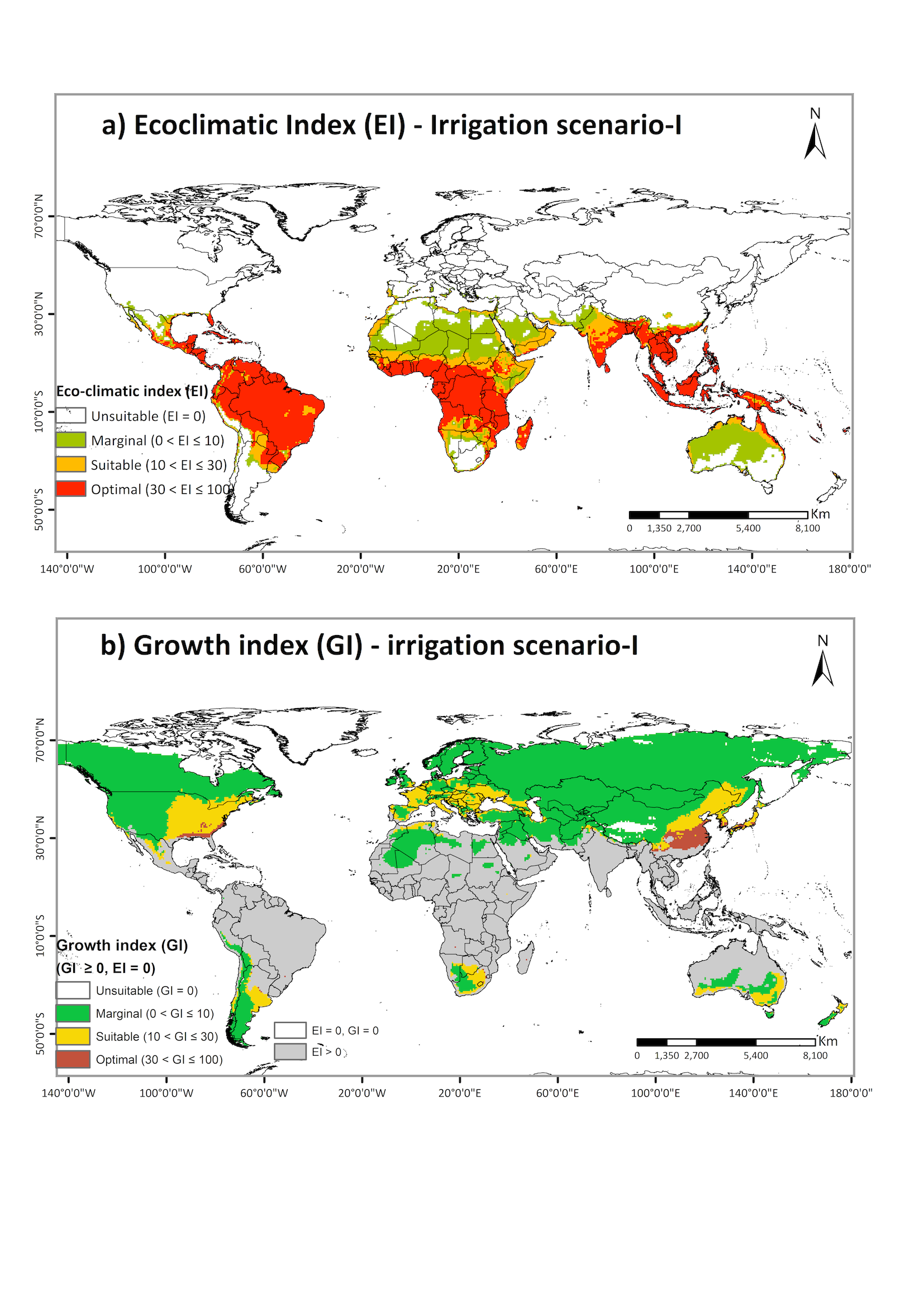


**Supplementary Fig. S3. The projected global climate suitability for FAW population establishment and seasonal population growth under historic climate using CLIMEX.** (a) Projected areas for year-round population establishment under irrigation-I scenario. (b) Growth index (GI) for seasonal population growth under irrigation-I scenario. Areas with EI > 0 support FAW year-round population establishment, areas with EI = 0 but GI > 0 support FAW seasonal population growth and areas with EI = 0 and GI = 0 are unsuitable for FAW survival. ArcMap 10.8 (https://desktop.arcgis.com/en/arcmap/).

**
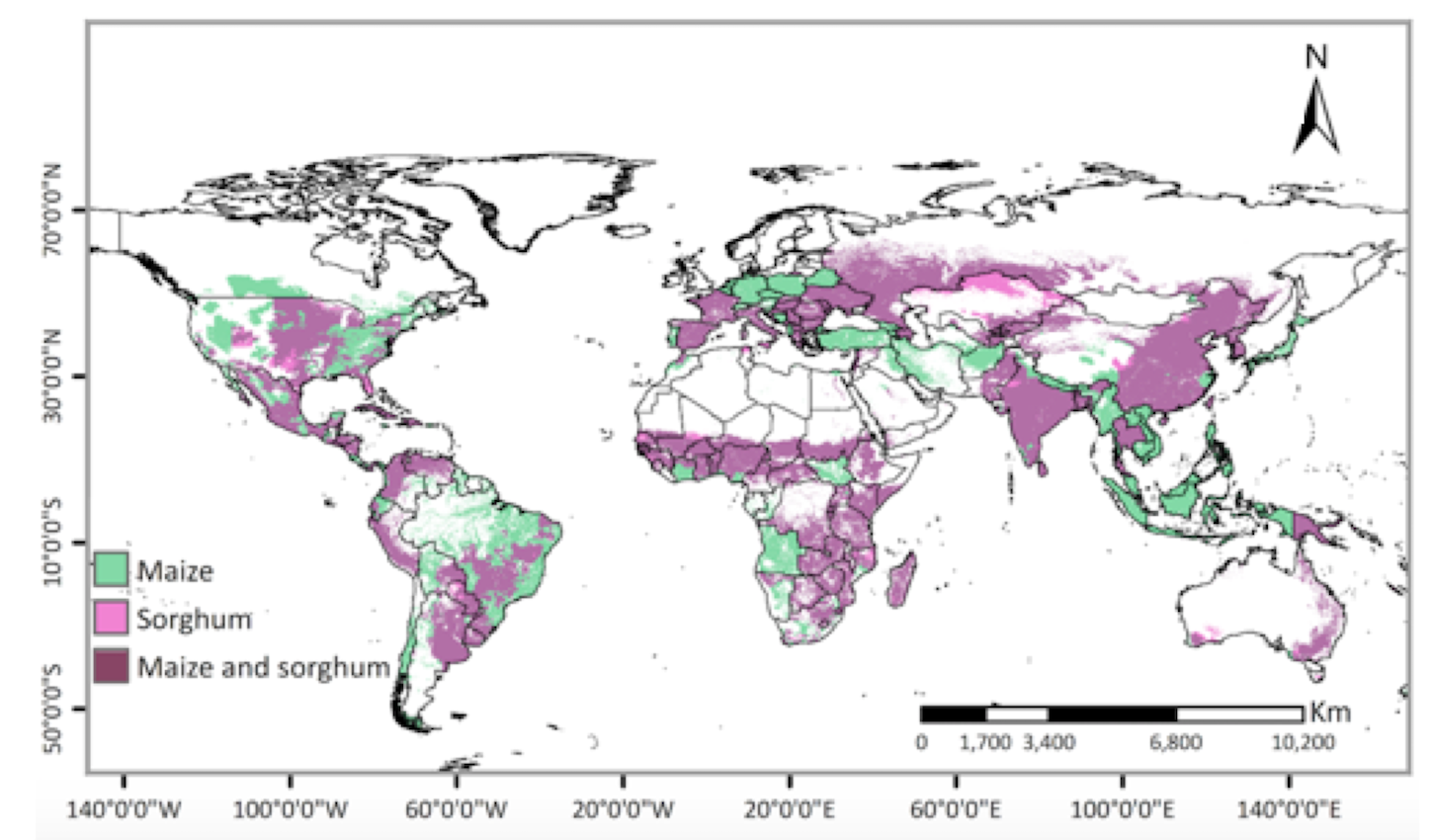
**

**Supplementary Fig. S4. Global distribution of maize and sorghum, two major host crops of FAW.** The map is based crop data obtained from the EarthStat database (<http://www.earthstat.org/>) created by Monfreda *et al.*^52^ ArcMap 10.8 (https://desktop.arcgis.com/en/arcmap/).

**
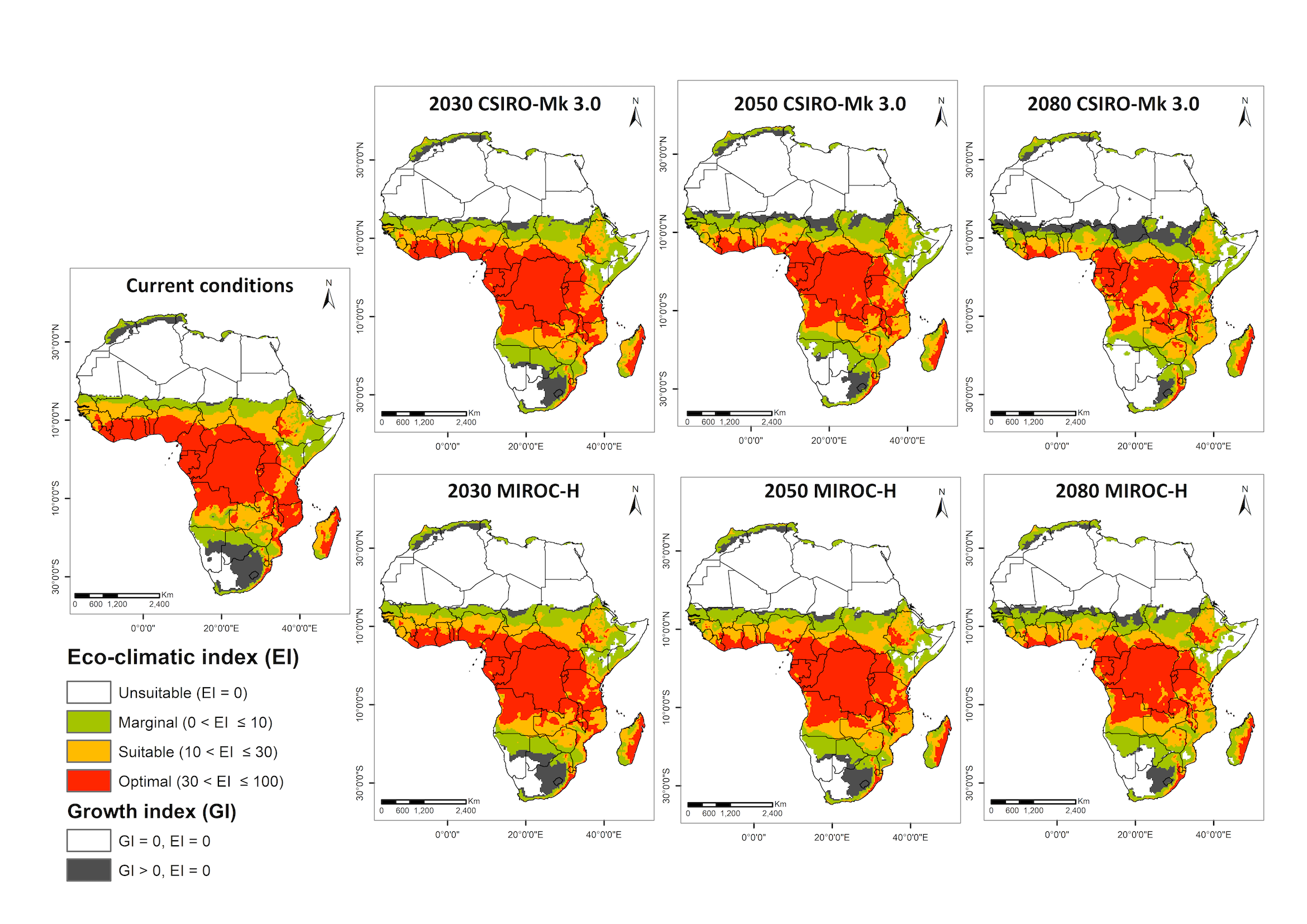
**

**Supplementary Fig. S5.** **The climatic suitability areas for FAW population establishment and seasonal population growth, considering rainfed conditions.** These projections were based on the current and projected future climates (2030, 2050 and 2080) under CSIRO-Mk3.0 (top) and MIROC-H (bottom) GCMs. Areas with EI > 0 support FAW year-round population establishment, areas with EI = 0 but GI > 0 support FAW seasonal population growth and areas with EI = 0 and GI = 0 are unsuitable for FAW survival. ArcMap 10.8 (https://desktop.arcgis.com/en/arcmap/).


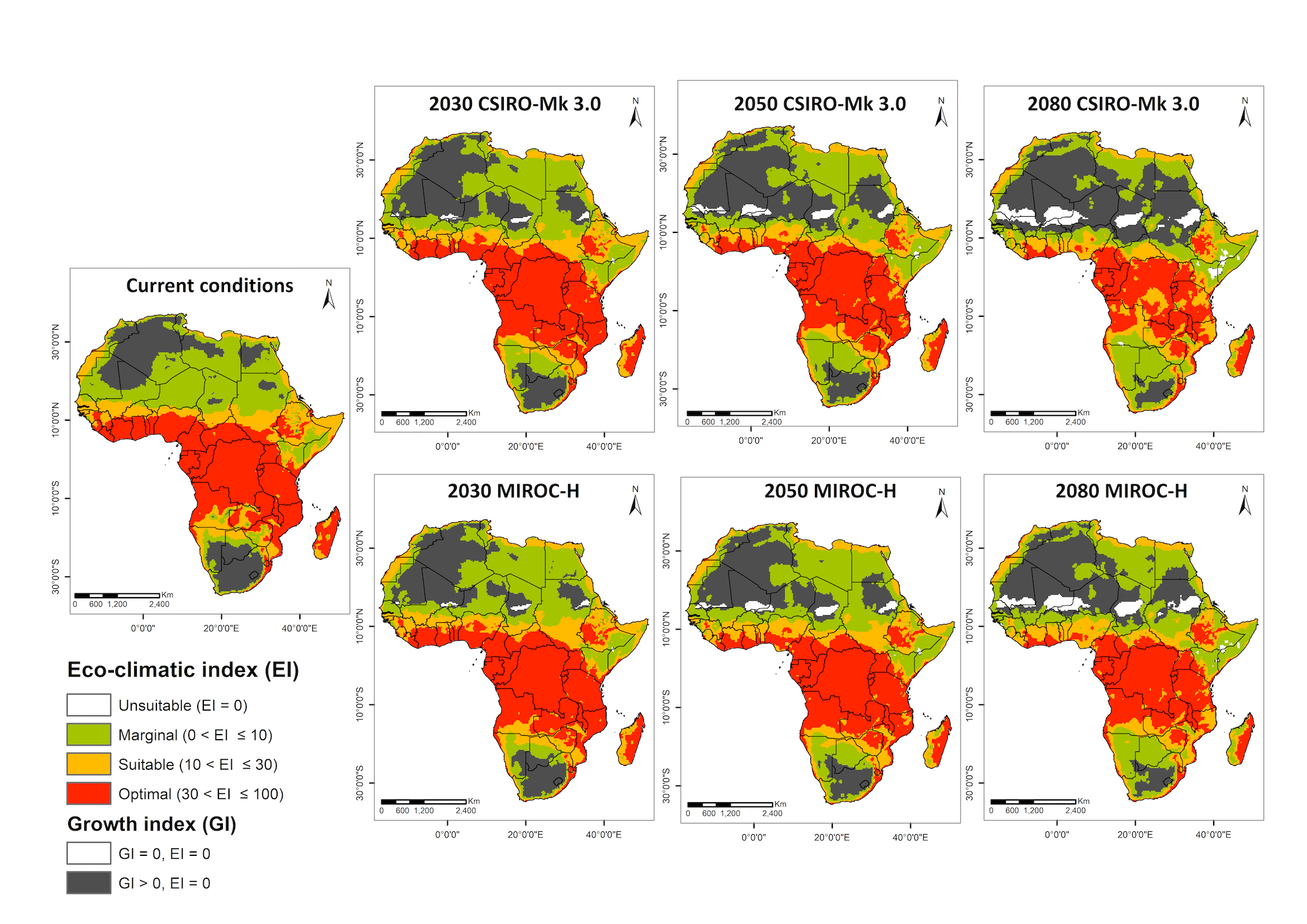


**Supplementary Fig. S6.** **The climatic suitability areas for FAW population establishment and seasonal population growth, considering irrigation scenario-I.** These projections were based on the current and projected future climates (2030, 2050 and 2080) under CSIRO-Mk3.0 (top) and MIROC-H (bottom) GCMs. Areas with EI > 0 support FAW year-round population establishment, areas with EI = 0 but GI > 0 support FAW seasonal population growth and areas with EI = 0 and GI = 0 are unsuitable for FAW survival. ArcMap 10.8 (https://desktop.arcgis.com/en/arcmap/).

**
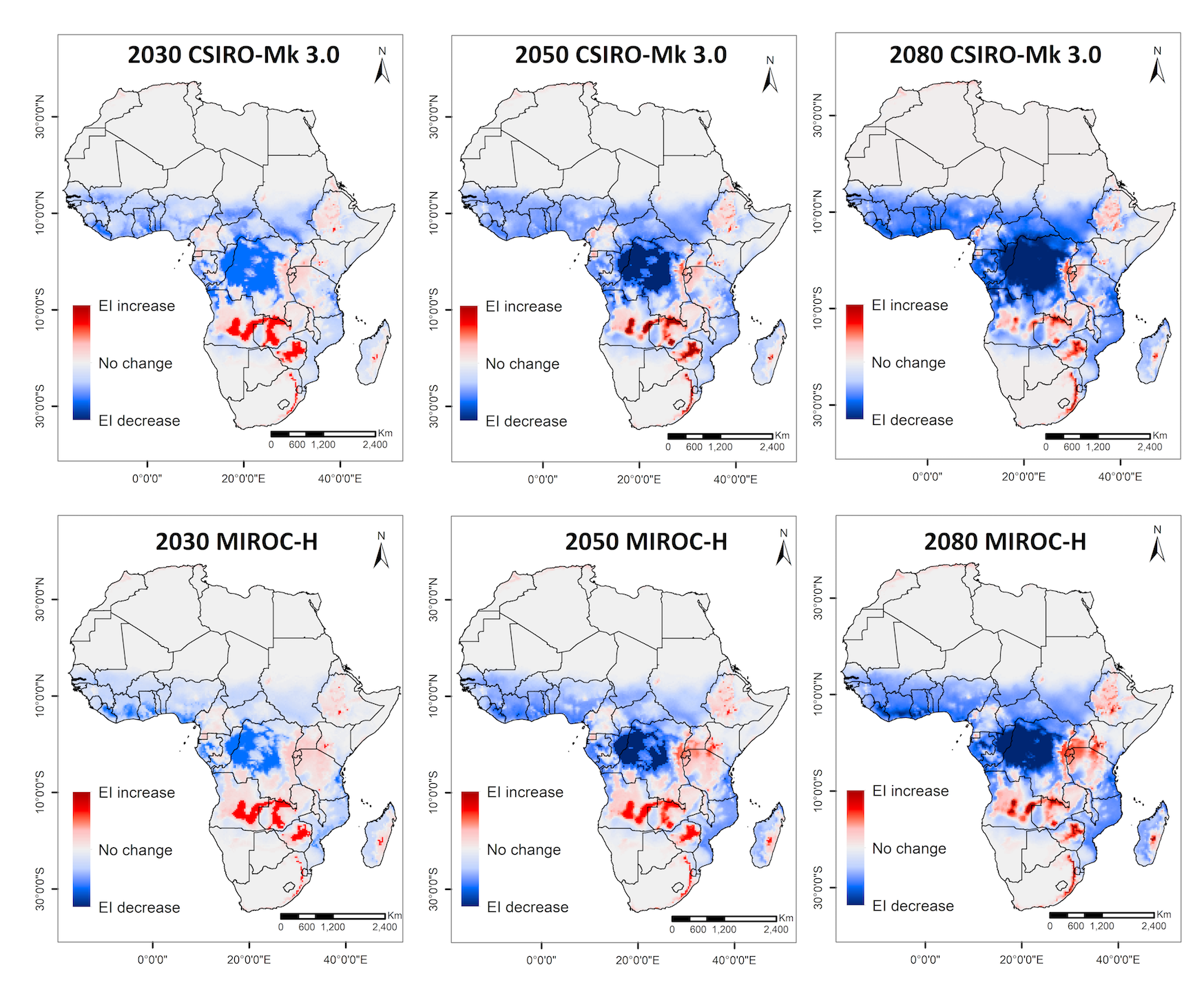
**

**Supplementary Fig. S7. Impact of climate change on climatic suitability area (EI) for FAW population persistence.** White indicates no change, red indicates an increase, and blue indicates decrease in EI values under the future projected climate from the current climate. Depth of color indicates the degree of changes in EI value. ArcMap 10.8 (https://desktop.arcgis.com/en/arcmap/).

**
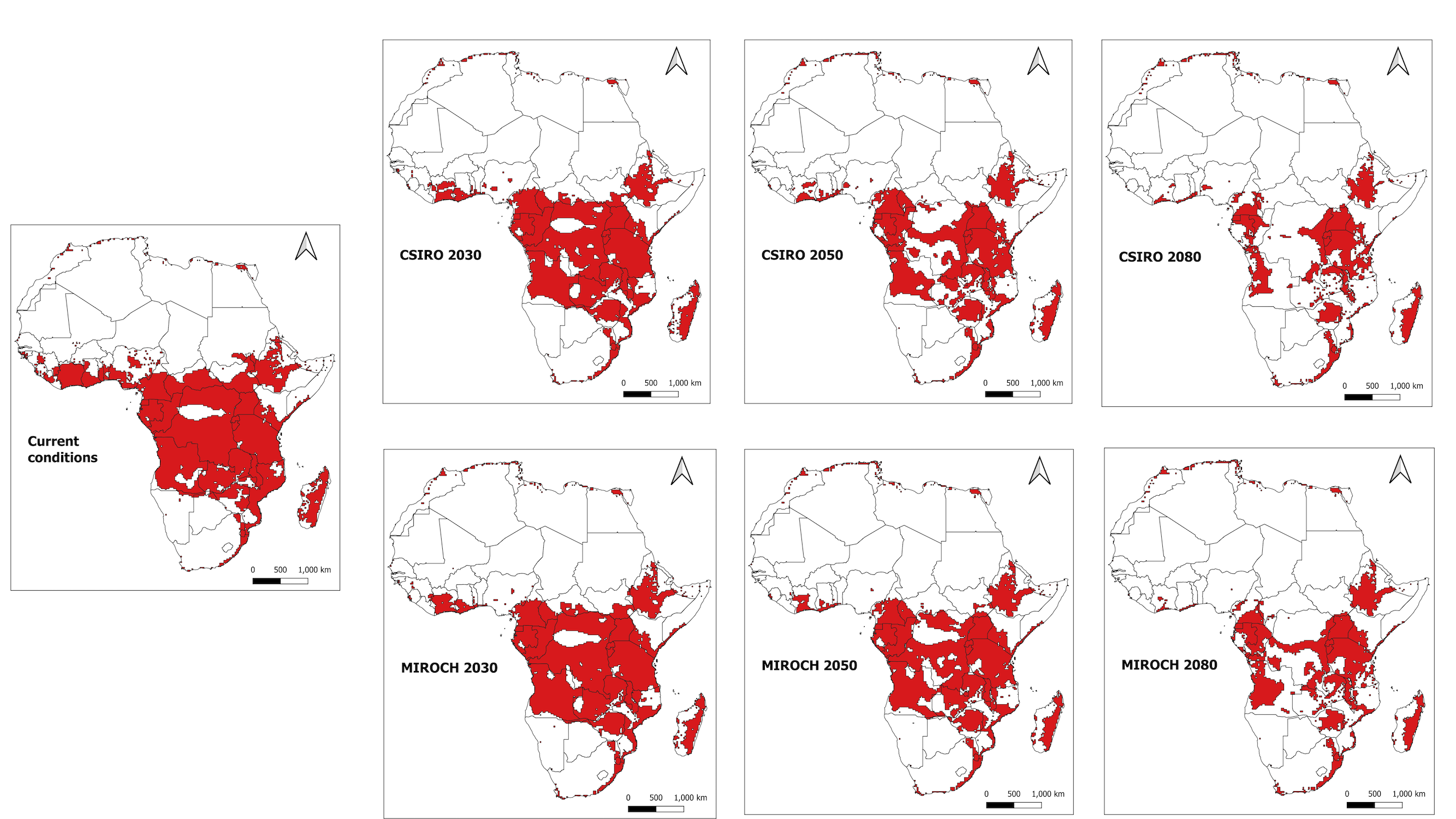
**

**Supplementary Fig. S8.** Potential overlap between FAW and maize, major host of FAW, under the current and projected future climates considering irrigation scenario-II. Red indicates areas with potential overlap between the pest and its host crop. ArcMap 10.8 (https://desktop.arcgis.com/en/arcmap/).


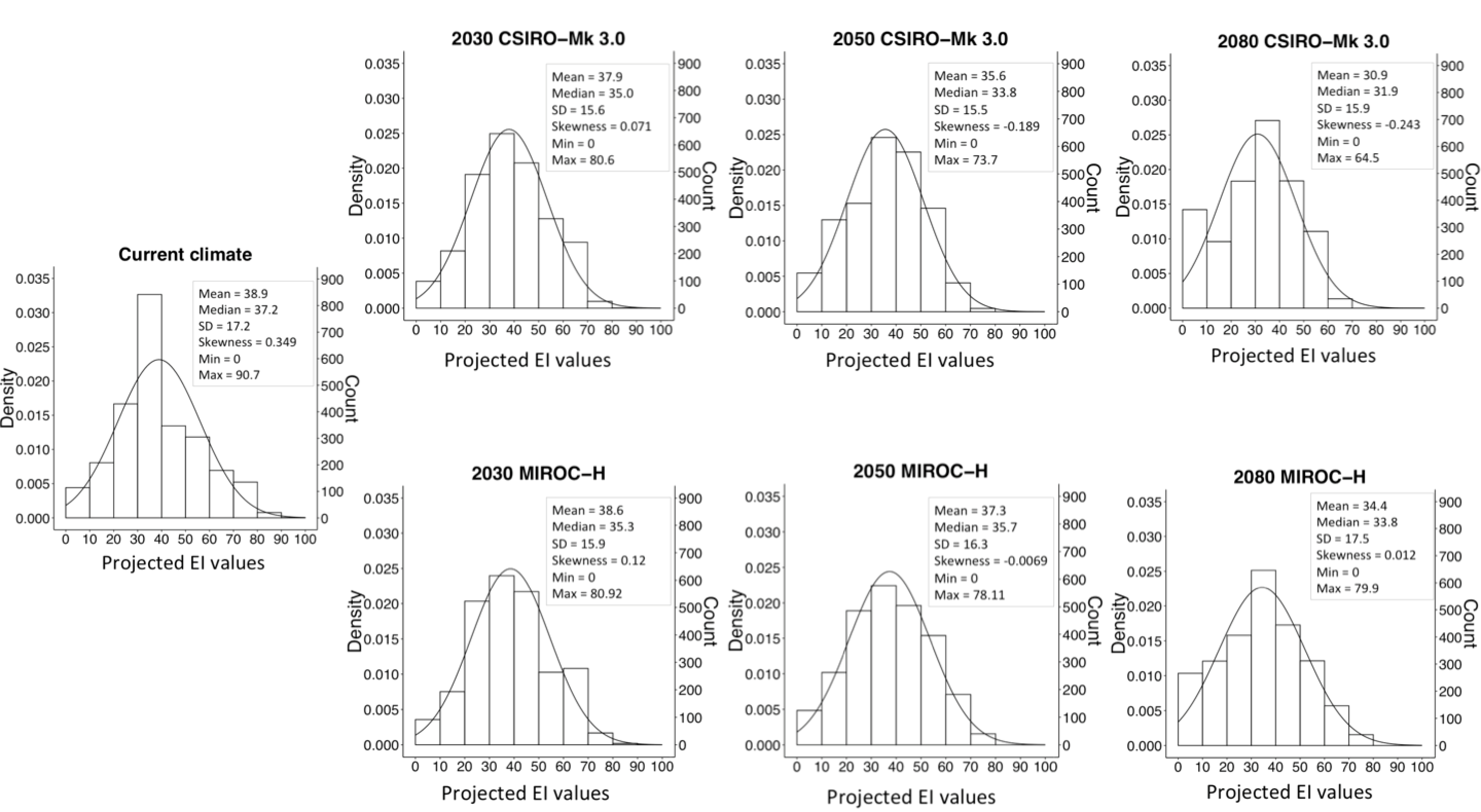


**Supplementary Fig. S9.**  Statistical analysis to evaluate the CLIMEX model performance.

**Supplementary Table S2:** CLIMEX parameter values used for modeling the distribution of maize (*Zea mays*).

| **Parameters** | | | **Value** |  |
| --- | --- | --- | --- | --- |
| **Temperature** | | |  |  |
| DV0 | Lower temperature threshold (˚C) | | 10 |  |
| DV1 | Lower optimal temperature (˚C) | | 18 |  |
| DV2 | Upper optimal temperature (˚C) | | 30 |  |
| DV3 | Upper temperature threshold (˚C) | | 35 |  |
| **Soil Moisture** | | |  |  |
| SM0 | Lower soil moisture threshold | | 0.1 |  |
| SM1 | Lower optimal soil moisture | | 0.7 |  |
| SM2 | Upper optimal soil moisture | | 0.9 |  |
| SM3 | Upper soil moisture threshold | | 1.3 |  |
| **Cold stress** | | |  |  |
| TTCS | Cold stress temperature threshold (˚C) | | 7 |  |
| THCS | Cold stress accumulation rate (week^-1^) | | -0.00007 |  |
| **Heat stress** | | |  |  |
| TTHS | Heat stress temperature threshold (˚C) | | 40 |  |
| THHS | Heat stress accumulation rate (week^-1^) | | 0.01 |  |
| **Dry stress** | | |  |  |
| SMDS | | Soil moisture dry stress threshold | 0.1 |  |
| HDS | | Dry stress accumulation rate (week^-1^) | -0.009 |  |
| **Wet stress** | | |  |  |
| SMWS | | Soil moisture wet stress threshold | 1.3 |  |
| HWS | | Wet stress accumulation rate (week^-1^) | 0.001 |  |
| **Irrigation** (mm day^-1^)* | | | | 2.5 |

The parameter values were adopted from previously conducted CLIMEX study on maize distribution^43^.

* This parameter was not used in the maize-CLIMEX model developed by Ramirez-Cabral *et al*^43^

**Supplementary Table S3:** CLIMEX parameter sensitivity values for *Spodoptera frugiperda* (FAW) parameters listed in Table 1, as applied to the CM10 1975H V1.2 global dataset under a rainfed conditions.

| Parameter | Mnemonic | Parameter range | | | Change in state variables | | | | | | | | | |
| --- | --- | --- | --- | --- | --- | --- | --- | --- | --- | --- | --- | --- | --- | --- |
|  |  | Low | Default | High | Range | Core dist’n | EI | GI | TI | MI | HS | CS | DS | WS |
| Cold Stress Temperature Threshold | TTCS | 7 | 8 | 9 | 1 | 0.4 | 2.9 | 0 | 0 | 0 | 0 | 17.15 | 0 | 0 |
| Limiting low moisture | SM0 | 0.05 | 0.15 | 0.25 | 0.8 | 0 | 1.6 | 2.3 | 0 | 10.72 | 0 | 0 | 0 | 0 |
| Limiting high temperature | DV3 | 35 | 36 | 37 | 0.3 | 0 | 1.6 | 1.7 | 4.2 | 0 | 0 | 0 | 0 | 0 |
| Dry Stress Threshold | SMDS | 0 | 0.1 | 0.2 | 0.3 | 6.7 | 1.8 | 0 | 0 | 0 | 0 | 0 | 43.27 | 0 |
| Cold Stress Temperature Rate | THCS | -0.006 | -0.005 | -0.004 | 0.2 | 0 | 0.7 | 0 | 0 | 0 | 0 | 4.58 | 0 | 0 |
| Lower optimal moisture | SM1 | 0.7 | 0.8 | 0.9 | 0.1 | 0 | 1.2 | 1.6 | 0 | 6.91 | 0 | 0 | 0 | 0 |
| Upper optimal temperature | DV2 | 29 | 30 | 31 | 0.1 | 0 | 4.3 | 4.4 | 6.7 | 0 | 0 | 0 | 0 | 0 |
| Heat Stress Temperature Threshold | TTHS | 38 | 39 | 40 | 0.1 | 0 | 0.4 | 0 | 0 | 0 | 12.26 | 0 | 0 | 0 |
| Upper optimal moisture | SM2 | 1.4 | 1.5 | 1.6 | 0 | 0 | 3 | 3.1 | 0 | 3.67 | 0 | 0 | 0 | 0 |
| Limiting high moisture | SM3 | 1.9 | 2 | 2.1 | 0 | 0 | 1.7 | 1.8 | 0 | 2.07 | 0 | 0 | 0 | 0 |
| Limiting low temperature | DV0 | 11 | 12 | 13 | 0 | 0 | 0.7 | 1.3 | 2.3 | 0 | 0 | 0 | 0 | 0 |
| Lower optimal temperature | DV1 | 24 | 25 | 26 | 0 | 0 | 2 | 2.2 | 4 | 0 | 0 | 0 | 0 | 0 |
| Heat Stress Temperature Rate | THHS | 0.002 | 0.0025 | 0.003 | 0 | 0 | 0.1 | 0 | 0 | 0 | 3.48 | 0 | 0 | 0 |
| Dry Stress Rate | HDS | -0.006 | -0.005 | -0.004 | 0 | 0.3 | 0.3 | 0 | 0 | 0 | 0 | 0 | 3.68 | 0 |
| Wet Stress Threshold | SMWS | 1.9 | 2 | 2.1 | 0 | 0.3 | 0.7 | 0 | 0 | 0 | 0 | 0 | 0 | 2.5 |
| Wet Stress Rate | HWS | 0.008 | 0.01 | 0.012 | 0 | 0 | 0.3 | 0 | 0 | 0 | 0 | 0 | 0 | 1 |
| Degree-days per Generation | PDD | 320 | 400 | 480 | 0 | 0 | 0 | 0 | 0 | 0 | 0 | 0 | 0 | 0 |

**Supplementary Table S4:** Number of FAW occurrence records in Africa that fall into each categories of eco-climatic index under rainfed conditions and irrigation scenario-II.

| **EI GI categories** | **Rainfed conditions** | **Irrigation scenario-II** |
| --- | --- | --- |
| Unsuitable (EI = 0, GI = 0) | 17 | 11 |
| Unsuitable (EI = 0, GI > 0) | 31 | 33 |
| Marginal (0 < EI ≤ 10) | 98 | 98 |
| Suitable (10 < EI ≤ 30) | 913 | 769 |
| Optimal (30 < EI ≤ 100) | 1532 | 1680 |
| Total records | 2591 | 2591 |

Note: Areas with EI = 0 is unsuitable for FAW year-round population establishment. These areas could support seasonal population if GI value is greater than 0.
